# Identifying pyroptosis-related genes as novel therapeutic targets in diabetic foot ulceration

**DOI:** 10.1186/s13098-025-01880-9

**Published:** 2025-08-01

**Authors:** Kang Liu, Lei Lei, Xin-lei Yang, Xin-he Zhang

**Affiliations:** Department of Burn and Plastic Surgery, Affiliated Chenggong Hospital of Xiamen University, Wenyuan Road No.96, Siming District, Xiamen, Fujian PR China

**Keywords:** Diabetic foot ulcers, Diabetic wound healing, Pyroptosis, Bioinformatics, Differentially expressed genes, Immune infiltration

## Abstract

Diabetic foot ulcer (DFU) is considered one of the most challenging chronic complications for individuals with diabetes, and its global incidence continues to rise, imposing a substantial burden on society. During the treatment of DFU, the locally intense inflammatory response induced by pyroptosis may adversely affect wound healing, making this phenomenon a focus of research. This study employed bioinformatics methods to systematically analyze the role of pyroptosis-related genes (PRGs) in the healing process of diabetic foot ulcers. Based on public datasets GSE147890 and GSE80178, we performed differential expression analysis, random forest, and LASSO regression to screen for key genes, and constructed and evaluated a multivariate logistic regression diagnostic model. We analyzed the GEO datasets GSE147890 and GSE80178, identifying 1336 and 2727 differentially expressed genes (DEGs), respectively. The intersection analysis with PRGs revealed 9 pyroptosis-related differentially expressed genes (PRDEGs). Functional enrichment analysis associated these genes with pathways such as I-kappaB kinase/nuclear factor-kappaB (IKK/NF-κB) signaling and mitophagy. Six key PRDEGs (FSTL1, PINK1, HDAC3, ULK1, CPTP, and NOD2) were selected, and a diagnostic model was constructed using random forest and LASSO regression. The accuracy of the model was assessed through multivariate logistic regression, calibration curve analysis, decision curve analysis (DCA), and receiver operating characteristic (ROC) curve analysis. The model demonstrated excellent diagnostic performance, with an area under the curve (AUC) of 1.000 in both the training and validation sets. This study highlights the importance of PRGs in diabetic wound healing (DWH). Our findings not only elucidate the mechanisms of action of PRGs in diabetic wound healing but also provide a theoretical basis for the development of clinical early diagnosis and individualized treatment strategies, holding significant clinical application potential. Future research should validate these findings in larger populations and explore therapeutic interventions targeting these pathways to improve DWH outcomes.

**Supplementary Information:**

The online version contains supplementary material available at 10.1186/s13098-025-01880-9.

## Introduction

Data show that there are approximately 463 million individuals with diabetes globally, and the number of patients will increase to 700 million by 2045 [1]. Diabetic foot ulcers (DFUs) are among the most debilitating complications for diabetic patients; they are the leading cause of nontraumatic lower limb amputations and significantly impact the physical and mental health of approximately 15–25% of the diabetic population. Diabetic wound healing is somewhat similar to normal wound healing; however, the hyperglycemic state in diabetic patients can lead to weakened local immune responses, reduced blood flow, and diminished cellular responsiveness, resulting in delayed wound healing [2]. The 2023 IWGDF (International Working Group on the Diabetic Foot) guidelines present updated strategies for managing diabetic wound healing (DWH), emphasizing a multidisciplinary team approach to develop personalized treatment plans. Conventional treatment methods include optimal blood glucose control, infection management, and surgical debridement. Novel therapies may encompass stem cell-based treatments, growth factor injections, ultrasound therapy, and negative pressure wound therapy, among others, with the aim of accelerating the wound healing process [3]. However, a subset of patients in clinical practice still does not achieve satisfactory outcomes, necessitating amputation to prevent the spread of infection. This highlights the critical requirement for the identification of novel therapeutic avenues to effectively cure diabetic foot ulcers and reduce the associated amputation rates [4].

In recent years, studies have found that the inflammatory response plays a crucial role during DFUs. Pyroptosis, as a novel form of programmed inflammatory cell death, may exacerbate this chronic inflammatory state [5]. Pyroptosis is characterized by the formation of cell membrane pores, the release of cellular contents, and the activation of inflammatory factors [6]. In diabetic wound healing, pyroptosis exacerbates the local inflammatory response by releasing a large amount of pro-inflammatory cytokines, such as IL-1β and IL-18 [7]. This excessive inflammation is a key factor contributing to the prolonged non-healing of diabetic wounds, as it inhibits the normal healing process. Modulating pyroptosis-related genes (PRGs) may become a new strategy to intervene in diabetic wound healing disorders, promoting wound healing by reducing the inflammatory response. Cell pyroptosis is triggered by gasdermin proteins, culminating in intracellular fluid accumulation, membrane lysis, and the release of proinflammatory agents [8, 9]. The pathways that regulate pyroptosis include the traditional caspase-1-dependent pathway, the alternative caspase-4/5/11-dependent pathway, and the caspase-3-mediated pathway [10]. Several studies have demonstrated the involvement of pyroptosis in diabetic complications such as diabetic nephropathy and retinopathy and that inhibiting pyroptosis can diminish the excessive secretion of inflammatory cytokines, thereby slowing the progression of disease [11]. Nevertheless, the precise function of PRGs in the process of DWH has yet to be significantly investigated. Given the inflammatory nature of DFUs, the aim is to modulate PRGs to inhibit the pyroptotic pathway and reduce the secretion of inflammatory chemokines, potentially slowing the progression of diabetic complications and enhancing the healing of diabetic wounds.

Recent advancements in bioinformatics have enabled the detailed examination of gene expression patterns to identify key regulatory genes and pathways involved in disease processes. This study aims to investigate the functions of key genes (PRGs) associated with DWH and to construct an effective diagnostic model to enhance the understanding of DWH. By integrating various bioinformatics analyses, we will identify biological pathways related to DWH and propose potential mechanistic hypotheses to elucidate the roles of the identified key genes in this process. This research not only elucidates the roles of PRGs in DWH but also provides a theoretical foundation for subsequent clinical applications and therapeutic strategies.

## Materials and methods

### Data acquisition

**Fig. 1:**
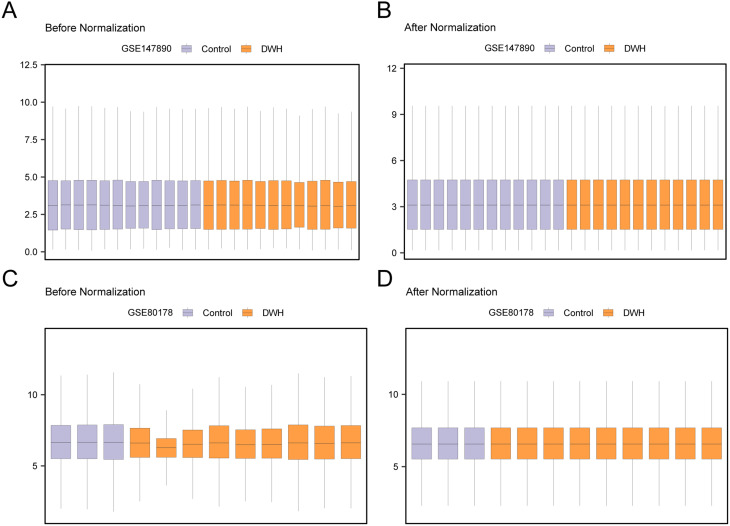
Data to batch processing (**A**) Box plot of GSE147890 distribution of dataset before batch execution. (**B**) The boxplot of GSE147890 distribution of the data collection after batch execution. (**C**) Boxplot of GSE80178 distribution of dataset before debatching. (**D**) The boxplot of GSE80178 distribution of the dataset after debatching. DWH, Diabetic Wound Healing. Orange is the DWH group, purple is the Control group. The y-axis represents the values of gene expression data, which are usually normalized or standardized expression levels

Through the R package GEOquery [12] (Version 2.70.0) from a GEO database [13] (https://www.ncbi.nlm.nih.gov/geo/) download DWH dataset (GSE147890 [14], GSE80178 [15]). The samples in GSE147890 are derived from bioengineered skin equivalents orthotopically transplanted into NMRI hairless mice (Rj: NMRI-Foxn1nu). These skin equivalents are composed of human keratinocytes (epidermal component) and viable human fibroblasts (dermal component), with the fibroblasts embedded in a fibrogenic gel matrix (*Homo sapiens*), and GSE80178 was from *Homo sapiens*. Both datasets were derived from Homo sapiens and foot skin tissue samples, ensuring biological comparability and enhancing the reliability of the analytical results. Concurrently, we rigorously removed batch effects by employing the R packages sva and limma for batch effect correction and data normalization, thereby ensuring the accuracy and reliability of the analysis. The related results are presented in the box plots (Fig. 1). The tissue source of GSE147890 was skin tissue, and the tissue source of GSE80178 was foot skin tissue. The chip platform of the GSE147890 dataset was GPL571, and the chip platform of the GSE80178 dataset was GPL16686. See Table 1 and Supplementary Table S1 for details. From dataset GSE147890, 12 DWH and 12 control samples were selected, whereas GSE80178 comprised 9 DWH and 3 control samples for this study.

**Table 1:** GEO microarray chip information

	GSE147890	GSE80178
Platform	GPL571	GPL16686
Species	Homo sapiens	Homo sapiens
Tissue	Skin Tissue	Foot Skin Tissue
Experiment Type	Expression profiling by array	Expression profiling by array
Abstract	This study developed a novel diabetic skin humanized mouse model with delayed wound healing and performed transcriptomic analysis to elucidate the molecular mechanisms.	This study compared genomic profiles of DFU and acute wounds in human skin, identifying miR-15b-5p as a major regulator leading to suboptimal inflammatory response and diminished DNA repair mechanisms in DFU.
Samples in DWH group	12	9
Samples in Control group	12	3
Reference	PMID: 33,396,192	PMID: 29,273,315

GEO, Gene Expression Omnibus; DWH, Diabetic Wound Healing; DFU, Diabetic foot ulcer

The GeneCards database [16] (https://www.genecards.org/) is a comprehensive collection of PRGs and offers extensive details about human genes. “Pyroptosis” was entered as the search term, and exclusively “protein-coding” genes related to pyroptosis were retained, resulting in the identification of 575 PRGs. Additionally, we used ‘Pyroptosis’ as a keyword to search for PRGs in scholarly publications found in the PubMed database (https://pubmed.ncbi.nlm.nih.gov/). Following the process of merging and eliminating duplicates, a final count of 582 PRGs was obtained, with comprehensive details presented in Supplementary Table S2.

To ensure the accuracy and comparability of data integration, we first performed visual assessments using box plots and Principal Component Analysis (PCA) to preliminarily identify potential batch effects across different datasets. For the detected batch differences, we employed the ComBat method from the R package ‘sva’ to correct for batch effects, aiming to eliminate systematic biases arising from sample origins and chip platforms. Subsequently, the normalized expression matrix was standardized using the limma package, followed by subsequent differential expression analysis. The detailed analytical workflow and the corresponding software versions are specified in the Methods section. Quality control results, visualized via box plots, indicated that the batch effects were largely mitigated. Considering the potential batch effects introduced by different sample origins and species, we conducted differential expression analysis separately for each dataset and intersected the differentially expressed genes to minimize batch effects. The datasets GSE147890 and GSE80178 underwent batch effect correction using the R package sva [17] (version 3.50.0). Furthermore, datasets GSE147890 and GSE80178 were standardized using the R package limma [18] (Version 3.58.1), and the annotation probes were subjected to standardization and normalization procedures. In this study, selected samples from the GSE147890 dataset were used as the test set, including 12 samples in the “DWH group” and 12 in the “control group,” which comprised 5 Control-0 h, 7 Control-24 h, 6 Diabetic-0 h, and 6 Diabetic-24 h samples. Samples from the control and diabetic groups at different time points were merged to comprehensively analyze gene expression changes during wound healing, thereby enhancing the understanding of diabetic wound healing. All samples from the GSE80178 dataset were used as the validation dataset for subsequent analyses, as detailed in Supplementary Table S3.

### Differential gene expression analysis

The datasets GSE147890 and GSE80178 were grouped into DWH and control groups. Variations in gene expression were analyzed through the limma R package (version 3.58.1), with|logFC| >0 and *P* < 0.05 as the criteria for identifying DEGs (differentially expressed genes). Genes with logFC > 0 and *P* < 0.05 were considered upregulated, whereas those with logFC < 0 and *P* < 0.05 were considered downregulated. The differential analysis results were visualized as a volcano plot via ggplot2 (version 3.4.4).

To identify PRDEGs (DEGs associated with pyroptosis) related to DWH, all DEGs meeting the criteria of|logFC| >0 and *P* < 0.05 from the differential expression analysis were selected from datasets GSE147890 and GSE80178 and interfaced with PRGs in all diabetic wound healing samples in datasets GSE147890 and GSE80178 to construct a Venn diagram. PRDEGs were obtained, and a heatmap was created with the R package pheatmap (version 1.0.12).

### Gene ontology (GO) and Kyoto encyclopedia of genes and genomes (KEGG) enrichment analyses

GO analysis [19] a technique for comprehensive functional annotation of gene sets, involves the examination of biological process (BP), cellular component (CC), and molecular function (MF) categories. KEGG [20], a notable resource, houses data on genetic pathways, diseases, and pharmaceuticals. Utilizing the R package clusterProfiler [21] (version 4.10.0), we executed enrichment analyses for GO terms and KEGG pathways on the PRDEGs. Statistical significance was determined by a cutoff of *P* < 0.05 and an FDR (q value) < 0.25.

### Gene set enrichment analysis (GSEA)

GSEA [22] was employed to evaluate gene set enrichment in the GSE147890 dataset via the R package clusterProfiler (v4.10.0), with genes ranked by logFC. The parameters included a seed number of 2020, 1000 permutations, and gene set sizes between 10 and 500. The analysis focused on *Homo sapiens* symbols in the c2 Canonical Pathways collection of MSigDB [23] (version Cp.All.V2022.1). For the GSEA analysis, utilizing the clusterProfiler R package (version 4.10.0), the significance threshold was set at a Benjamini–Hochberg corrected adj. *P* < 0.05, alongside a requirement for FDR (False Discovery Rate) < 0.25. DWH samples from GSE147890 were sorted into high- and low-risk groups according to the median risk score, and the limma package was used to analyze differential gene expression (v3.58.1). Genes with|logFC| >0 and *P* < 0.05 were subjected to GSEA with the same parameters, and the c2 collection of MSigDB was used for enrichment analysis, adhering to the same significance criteria.

### Screening of key genes

To identify pivotal genes and construct diagnostic protocols, we initially performed univariate logistic regression on PRDEGs, selecting those with *P* < 0.05. These genes were subsequently input into a random forest (RF) analysis. We employed RF and LASSO regression methods for screening PRDEGs. During model parameter optimization and feature selection, five-fold ten-repeat cross-validation was implemented to assess model robustness and prevent overfitting. The optimal parameters for both RF and LASSO regression were determined based on the cross-validation results. Using the randomForest package [24]we constructed a model with parameters set to seed (234) and ntree = 300, utilizing the expression matrix from the GSE147890 dataset. Variable importance was evaluated using the Mean Decrease in Gini (MDG) coefficient, and the most significant variables were selected via five-fold ten-repeat cross-validation. This approach aimed to balance bias and the adequacy of training data. Subsequently, we performed Least Absolute Shrinkage and Selection Operator (LASSO) [25] regression on the RF-screened results using the glmnet [26] package (version 4.1–8), setting seed (300) and running it for 200 iterations to prevent overfitting. LASSO regression, which includes a penalty term in linear regression, was visualized through diagnostic plots. The PRDEGs retained in the final LASSO model were deemed key genes for further analysis. Furthermore, the GSE80178 dataset was utilized as an external validation cohort to independently assess the model’s generalization ability and diagnostic performance.

### Key genes used to construct the diagnostic logistic regression model

We utilized a multivariate logistic regression model incorporating all identified key genes to estimate coefficients, which were then multiplied by gene expression levels to compute a risk score for each sample. The samples were sorted into high- and low-risk groups according to the median risk score, which was computed with the following equation:

$$\eqalign{ \>{\rm{Risk}}Score\> = & \>\sum {{\>_i}} Coefficient\>\left( {gen{e_i}} \right) \cr & * \>mRNA\>Expression\>\left( {gen{e_i}} \right) \cr} $$

We constructed a nomogram [27] via the rms package (v6.7-1) to graphically represent the relationships between key genes in the multivariate logistic model. Calibration analysis was then applied to generate a calibration curve, which was used to evaluate the model’s accuracy and discrimination. Decision curve analysis (DCA) [28] was conducted with ggDCA (v1.1) to analyze the clinical application of the key genes. Receiver operating characteristic (ROC) curves and area under the curve (AUC) values were computed via pROC (v1.18.5) to evaluate the diagnostic performance of the risk score in predicting the DWH, with AUCs ranging from 0.5 (low accuracy) to 1 (high accuracy).

### Verification of the differential expression of key genes

We visualized differences in the expression of key genes between the DWH cohort and controls in the GSE147890 and GSE80178 datasets. The pROC package (v1.18.5) was used to plot ROC curves and compute AUC values, which were used to assess the diagnostic impact of key gene expression on DWH occurrence. GO [9] annotation semantic analysis was conducted via GOSemSim [29] (v2.28.0) to evaluate functional correlations among key genes, which were then visualized as ‘Friends’. RCircos [30] (v1.2.2) was employed to depict the chromosomal locations of these genes.

### Immune infiltration analysis of key genes

ssGSEA [31] (single-sample gene set enrichment analysis) was used to assess the abundance of various immunocyte subsets, including activated CD8 + T cells, activated dendritic cells, gamma-delta T cells, etc. The enrichment scores from this analysis were used to create an immune cell infiltration matrix, which was then analyzed to identify significant differences between groups. Spearman’s correlation algorithm and the R package pheatmap (version 1.0.12) were employed to illustrate correlations among immunocytes. Similarly, the associations between key genes and immunocytes were calculated via Spearman’s algorithm, with significance at *P* < 0.05 represented in a correlation bubble chart constructed via the ggplot2 package within R (version 3.4.4).

### mRNA‒miRNA and mRNA‒TF interaction network analysis of key genes

The ENCORI (Encyclopaedia of RNA Interactomes) database [32] (v3.0) was utilized to predict miRNAs that interact with key genes on the basis of RNA interaction data, with a pancancerNum > 12 cutoff. Similarly, CHIPBase [33] (ChIP-seq Transcription Factor-Binding Site Database) version 3.0 was employed to identify TFs that bind to key genes via ChIP-seq data, which were filtered by a sum of upstream and downstream sample counts > 6. The mRNA‒miRNA and mRNA‒TF interaction networks were visualized via Cytoscape.

### Tissue collection and ethics statement

In this investigation, 10 participants were included, with 5 patients with diabetic foot ulcers (3 females, 2 males, age: 64.4 ± 8.4 years) in the DFU group and 5 patients with acute open fractures (3 females, 2 males, mean age: 60.2 ± 13 years) in the control group. For tissue sampling, in the DFU group, full-thickness skin tissue surrounding the diabetic wound was collected during the initial debridement. For the control group, full-thickness skin tissue surrounding the acute wound was collected during the initial debridement. The inclusion criterion for patients was Wagner grade 3 or higher diabetic foot ulcers (DFUs). For the control group, patients with acute open injuries were included only if the injury occurred within 6 h. No participants had received recent antibiotic or anti-inflammatory treatment, and no surgeries related to the wound were performed. The exclusion criteria were systemic diseases affecting healing, immunodeficiency, and smoking history. Detailed information on the patients is provided in Supplementary Table S4. All participants provided informed consent, and this study was approved by the Ethics Committee of the Affiliated Success Hospital of Xiamen University (Ethics Number: 73JYY2024145244).

### Quantitative PCR (qPCR)

Total RNA was isolated from skin tissue samples via TRIzol reagent (Invitrogen, 15596026) and subsequently purified through DNase treatment, followed by column purification with a RNeasy kit (Qiagen, 74106). cDNA synthesis was carried out using 1 µg of RNA and an RT reaction mixture (Promega, A3500). qPCR was performed on an I-Cycler and IQ real-time PCR system (Bio-Rad) with SYBR Green I. Gene expression was quantified via the 2DDCT method, with *GAPDH* used as the reference gene. The primer sequences are detailed in Supplementary Table S5.

### Explanation of variables

In this study, to ensure the accuracy and reliability of data analysis, we employed specific terminology to describe different variables. Here, “mod” refers to the model we constructed, which is used to describe the differences in gene expression and their correlation analysis between DWH samples and control samples. During the data analysis process, we employed logistic regression and random forest methods to screen for Key Genes, thereby establishing a predictive model. The construction of the model constitutes the core component of the entire analysis, aiding our understanding of the role of key genes in the process of diabetic wound healing.

“Batch” refers to batch effects introduced during sample processing. During the process of data processing and merging, considering that samples may originate from different time points or environmental conditions, systematic errors may be introduced, thereby affecting the reliability of gene expression data. Therefore, we utilized the ComBat function from the R package “sva” to perform batch effect removal on the two datasets, eliminating these batch effects to minimize their interference with subsequent analysis results. This step is crucial for ensuring the validity of the analytical outcomes, allowing for a more precise evaluation of the genuine changes in gene expression during DWH.

### Statistical analysis

Data analysis was performed using R software (v4.2.2). For comparisons of continuous variables between groups, independent samples t-tests were applied for normally distributed data, while Mann-Whitney U tests were used for non-normally distributed data. The Kruskal-Wallis test was employed for comparisons involving three or more groups. Spearman’s correlation analysis was conducted to assess molecular correlations. All *P* values were two-tailed, with statistical significance set at *P* < 0.05, unless otherwise specified. For multiple testing, *P* values were adjusted using the Benjamini-Hochberg method.

## Results

### Overview of the technical roadmap

**Fig. 2:**
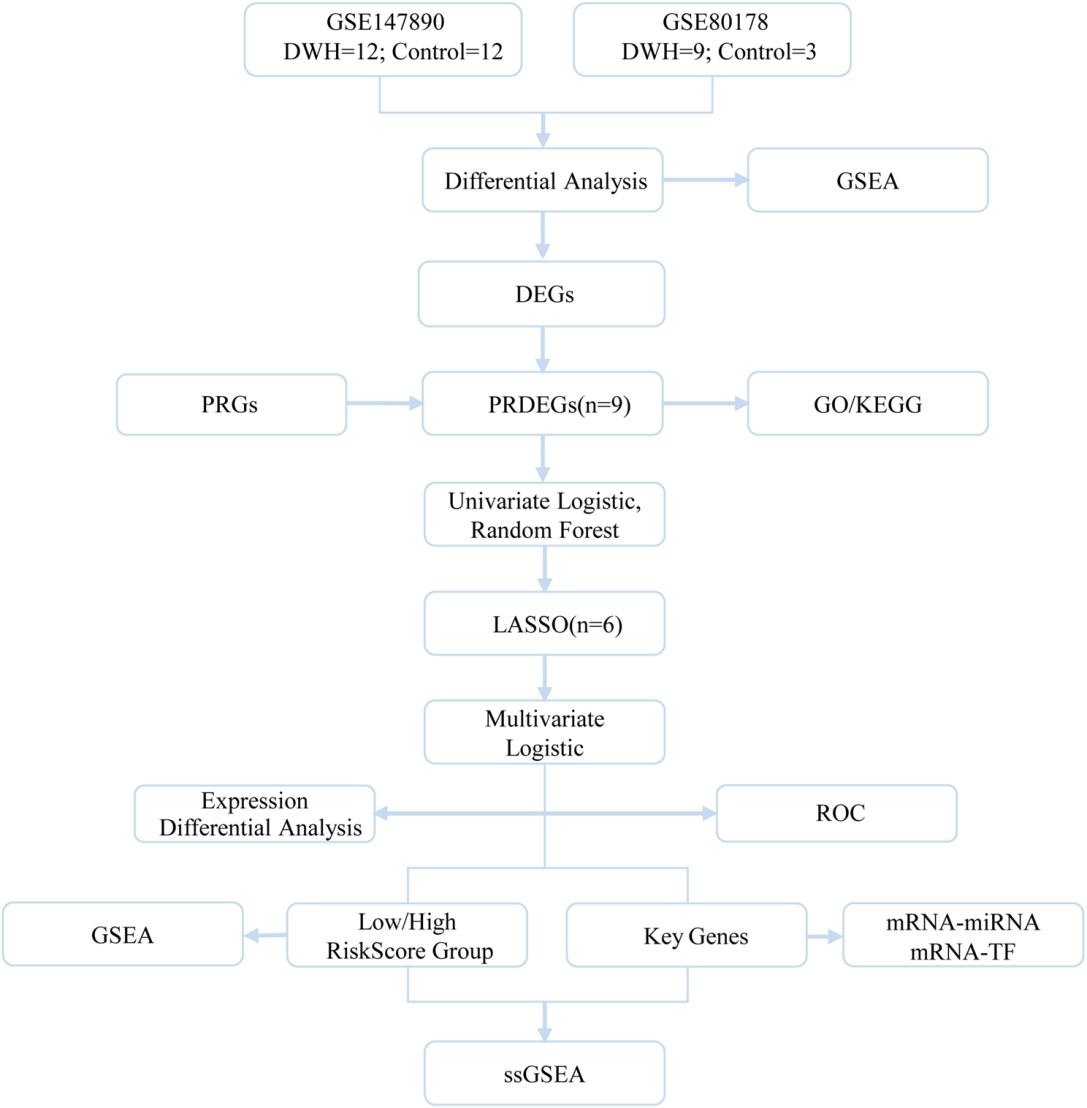
Technology roadmap

Analysis of DEGs and PRGs. DEGs were identified through comparative transcriptomic analysis. This figure highlights PRGs and their differentially expressed counterparts (PRDEGs) to elucidate their biological roles. Gene set enrichment analysis (GSEA) was conducted to identify enriched pathways, with functional annotations derived from Gene Ontology (GO) and Kyoto Encyclopedia of Genes and Genomes (KEGG). Least absolute shrinkage and selection operator (LASSO) regression was applied for feature selection, and the involvement of transcription factors (TFs) was examined. Furthermore, single-sample gene set enrichment analysis (ssGSEA) was performed to evaluate pathway activity in individual samples (Fig. 2).

### Processing of the diabetic wound healing dataset

Box plots (Supplementary Figs. 2 A, B) were employed to assess the variations in GSE147890 expression data before and after batch effect correction, whereas additional box plots (Supplementary Figs. 2 C, D) facilitated a similar comparison for GSE80178. The box plot analyses indicated that the batch bias within the DWH dataset was largely mitigated following the removal process.

### Differentially expressed genes related to pyroptosis in diabetic wound healing

**Fig. 3:**
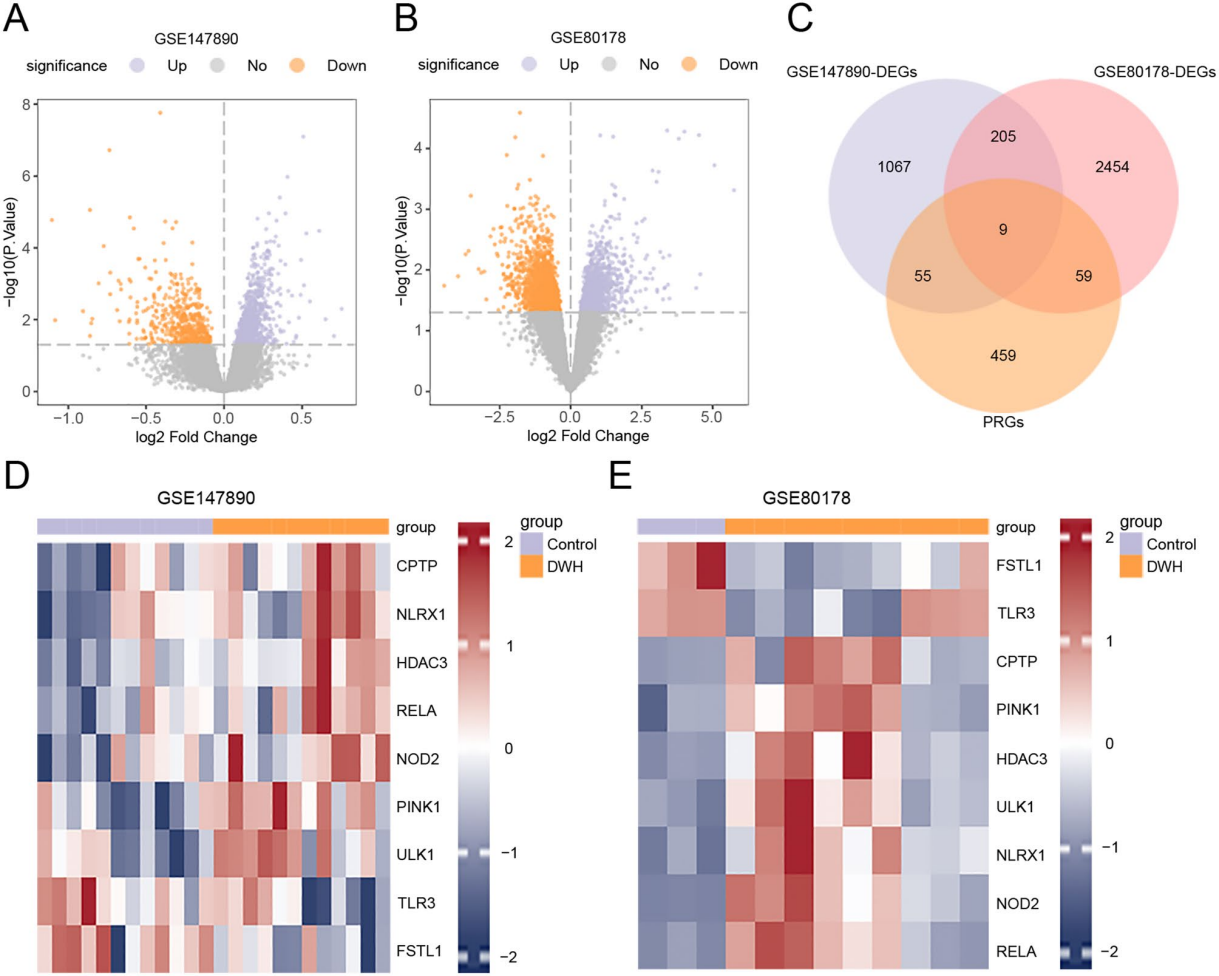
Differential gene expression analysis. **A**-**B**. Volcano plots depicting DEGs in the GSE147890 (**A**) and GSE80178 (**B**) datasets, comparing the DWH and control groups. **C.** Venn diagram showing the overlap of DEGs and PRGs in diabetic wound healing samples from both datasets. **D**-**E**. Heatmaps illustrating PRDEGs associated with pyroptosis in GSE147890 (**D**) and GSE80178 (**E**). Colors: The DWH group is shown in orange, and the control group is shown in purple; in heatmaps, red indicates high expression, and blue indicates low expression.

Variations in gene expression, analyzed through the limma R package, revealed 1336 DEGs in GSE147890 (820 upregulated, 516 downregulated) and 2727 DEGs in GSE80178 (1277 upregulated, 1450 downregulated), with|logFC| >0 and *P* < 0.05, as shown in volcano plots (Figs. 3A, B). To identify PRDEGs, DEGs from both datasets were intersected with PRGs, yielding 9 PRDEGs: *PINK1*, *FSTL1*, *ULK1*, *HDAC3*, *NOD2*, *CPTP*, *TLR3*, *RELA* and *NLRX1* (Fig. 3C). Comparative expression patterns of DEGs across sample groups in GSE147890 and GSE80178 were visualized via a heatmap generated via the R package pheatmap (Figs. 3D, E).

### Analysis of gene ontology and KEGG pathway enrichment

**Fig. 4:**
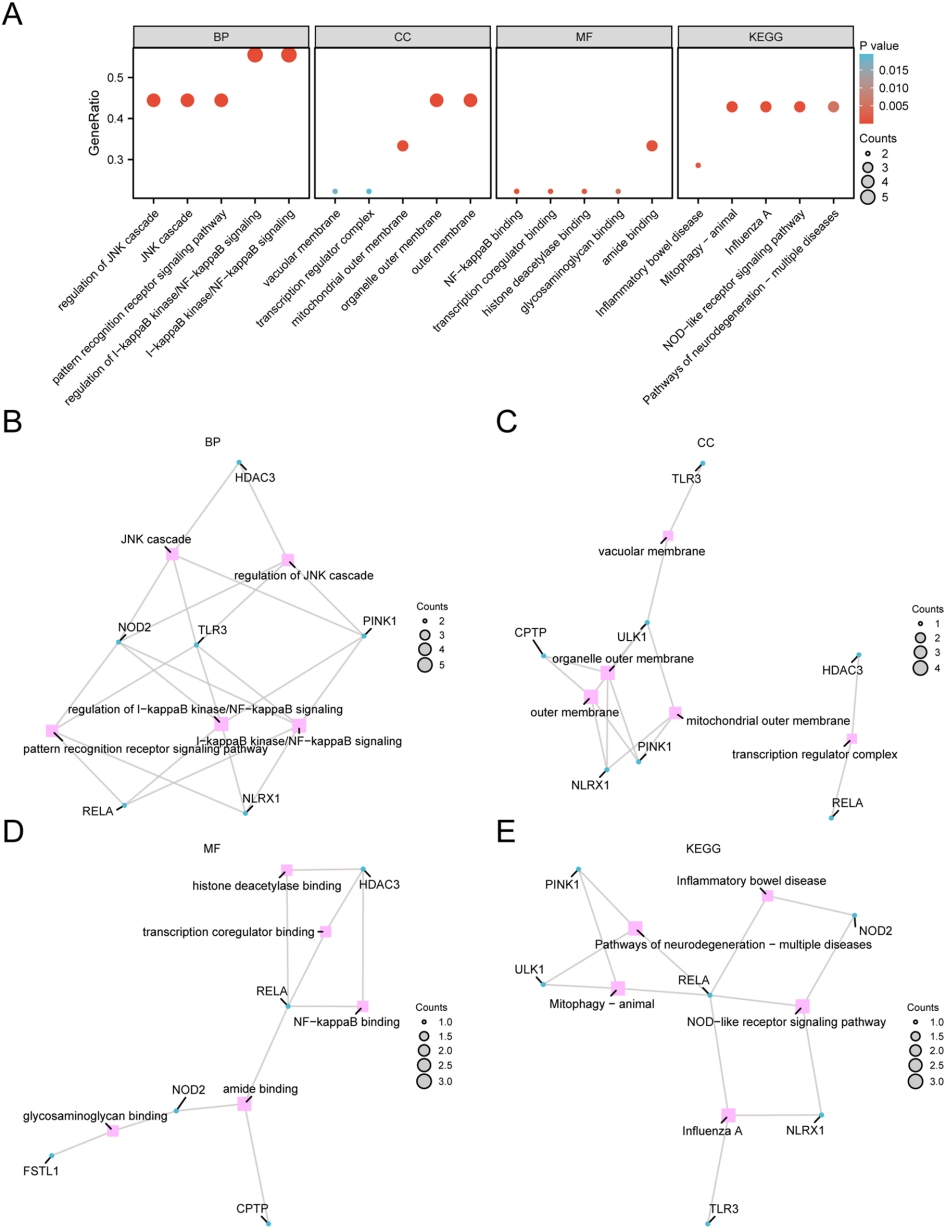
GO and KEGG enrichment analysis of the PRDEGs. **A**. Bubble chart depicting GO and KEGG https://www.kegg.jp/kegg/kegg1.html/ [20], enrichment for PRDEGs, including BP, CC, MF, and biological pathways. The GO and KEGG terms are plotted on the x-axis. The bubble size indicates the number of genes, and the color represents the P value, with red indicating smaller P values and blue indicating larger P values. **B**-**E**. Network diagrams for GO (**B**-**D**) and KEGG (E) enrichment, with pink nodes for items, blue nodes for molecules, and lines depicting their relationships. BP, Biological Process; CC, Cellular Component; MF, Molecular Function. Enrichment analysis was conducted with P < 0.05 and an FDR (q value) < 0.25.

The 9 PRDEGs were subjected to enrichment analyses, and the detailed outcomes are presented in Supplementary Table S6. These findings indicate that these PRDEGs are predominantly involved in modulating the I-kappaB kinase/NF-kappaB signaling (IKK/NF-κB) pathway in DWH. IKK/NF-κB pathway, regulation of the JNK cascade, JNK cascade and pattern recognition receptor signaling pathway (BP); organelle outer membrane, outer membrane, mitochondrial outer membrane, vacuolar membrane and transcription regulator complex (CC); amide binding, NF-kappaB binding, transcription coregulator binding, and molecular functions (MFs), such as histone deacetylase binding and glycosaminoglycan binding. It was also enriched in mitophagy-animal, influenza A, and *NOD*-like receptor signaling pathways and in the biological pathways of neurodegeneration-multiple diseases and inflammatory bowel disease (KEGG). The enrichment analysis results are depicted with bubble plots (Fig. 4A). Network diagrams illustrate BP, CC, MF, and KEGG biological pathways, with node size proportional to the number of molecules in each category (Figs. 4B-E). Connections denote molecule annotations and relevance.

### GSEA

**Fig. 5:**
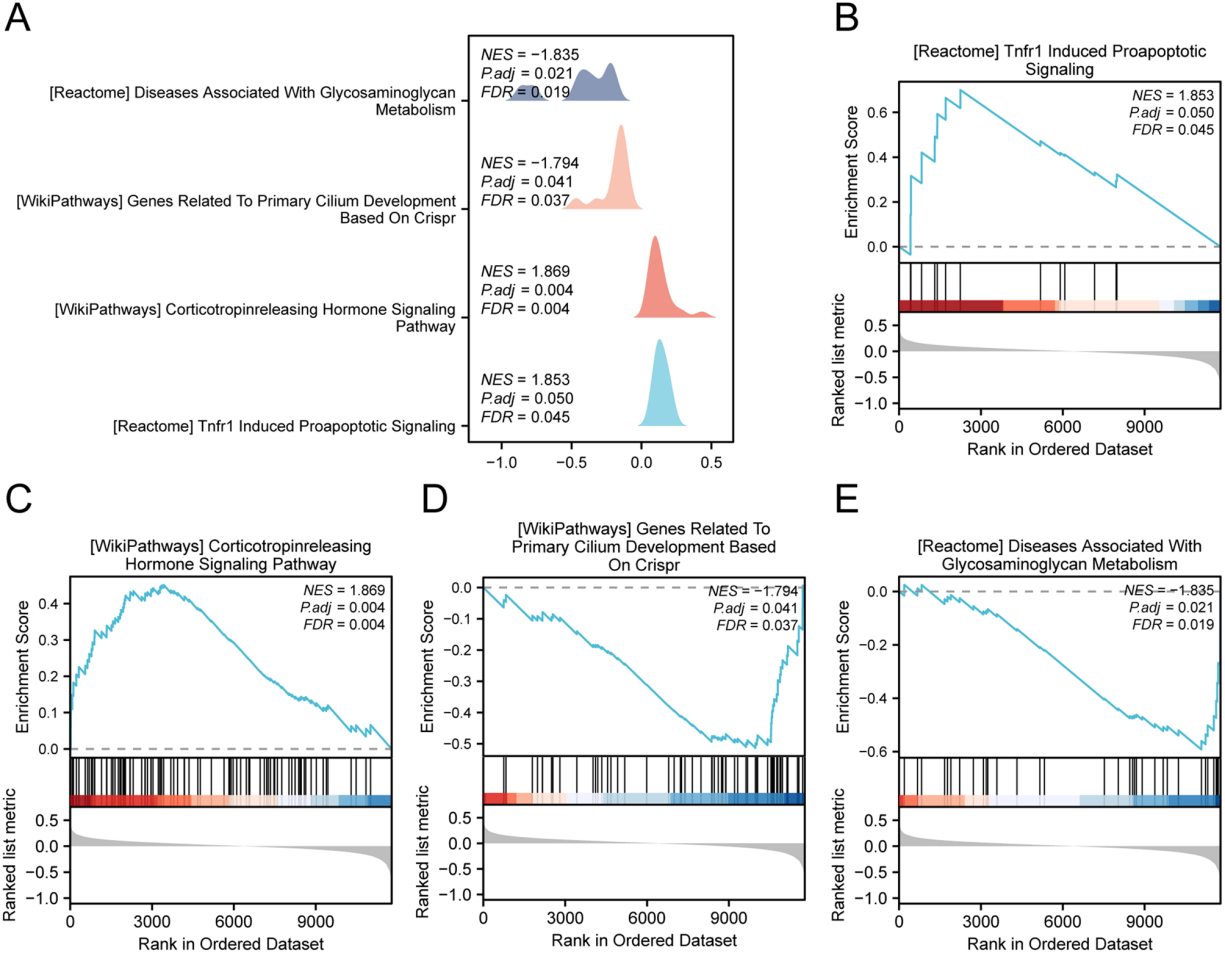
GSEA of diabetic wound healing **A**. Mountain plot showing four enriched biological functions identified via GSEA of the GSE147890 dataset. **B**-**E**. GSEA revealed significant enrichment of the genes encoding Tnfr1-induced proapoptotic signaling (**B**), the corticotropin-releasing hormone signaling pathway (**C**), genes related to primary cilium development based on CRISPR (**D**), and diseases associated with glycosaminoglycan metabolism (**E**). Significance was assessed with adj. P < 0.05 and FDR < 0.25, with P values corrected by Benjamini‒Hochberg (**BH**)

To assess the entire gene expression profile in the GSE147890 dataset for DWH, GSEA was employed to analyze BP, CC, and MF. The results are presented in Fig. 5A and Supplementary Table S7. Notably, genes in GSE147890 were significantly enriched in Tnfr1-induced proapoptotic signaling, the corticotropin-releasing hormone signaling pathway, genes related to primary cilium development based on CRISPR, diseases associated with glycosaminoglycan metabolism, and other relevant functions and pathways (Figs. 5B-E).

### Screening of key genes

**Fig. 6:**
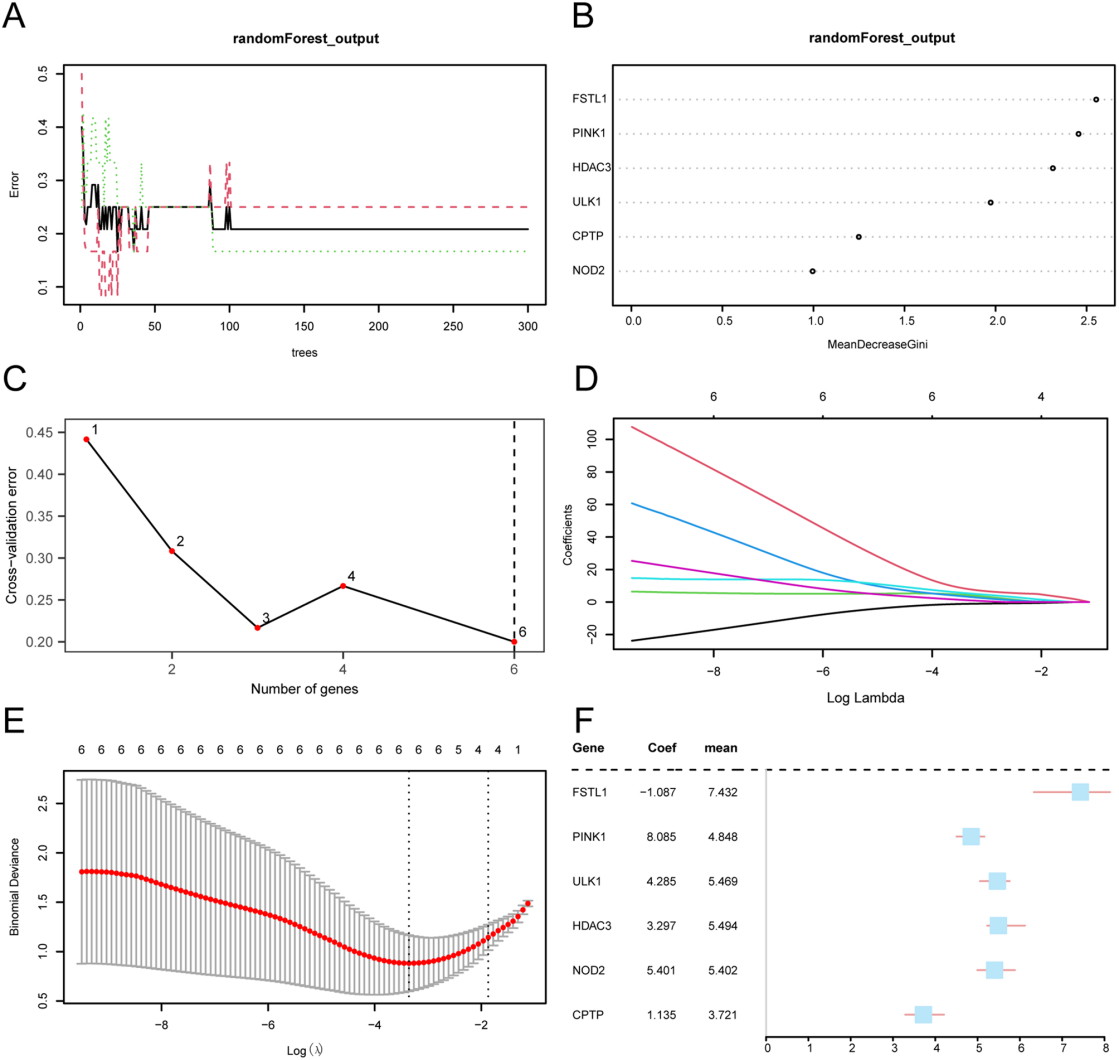
Key gene screening. (**A**) RF training error: Trees (0–300) vs. error rate. Black line: training error; red line: test error. (**B**) PRDEGs MDG Scatter: MDG on the x-axis shows gene importance for DWH vs. control. Genes sorted by MDG. (**C**) Cross-validation error: gene count on the x-axis and cross-validation error on the y-axis. (**D**) LASSO regression trace: Log lambda on the x-axis and gene regression coefficients on the y-axis. Lines show gene importance. (**E**) LASSO Regression Diagnostic: Bremner’s Bias vs. Log(lambda). Red line: optimal lambda. (**F**) LASSO Key Gene Forest: Summarizes selected genes (FSTL1, PINK1, ULK1, HDAC3, NOD2, CPTP) with coefficients. The coefficient size indicates the diagnostic power for DWH

We screened the 9 PRDEGs for diagnostic potential in the DWH via univariate logistic regression and identified those with *P* values < 0.05 (Supplementary Table S8). We then adopt the RF algorithm for the 9 PRDEGs in the GSE147890 dataset (DWH/Control) to analyze the expression quantity groups. The decision tree error curve was drawn by setting the number of seeds to 234 and the number of decision trees to 300 (Fig. 6A). The findings indicated that the error reached the lowest value and tended to be stable when the number of decision trees was approximately 100. We plotted the mean decrease Gini (MDG) scatter plot (Fig. 6B) for the 9 PRDEGs to identify important genes. The MDG measures the average reduction in impurity of nodes in the tree, with higher values indicating greater importance in distinguishing between the DWH and control groups. We then selected the number of genes by performing five tenfold cross-validations and generating a cross-validation error curve (Fig. 6C). The figure shows that when the number of genes is 6, the error of the model is the smallest, and when the model is combined with MDG, the specific genes are selected for subsequent analysis. According to the results (Figs. 6B, C), the algorithm screens out 6 PRDEGs, which are important for the diagnosis of DWH, according to importance degree from large to small, in the following order: *FSTL1*, *PINK1*, *HDAC3*, *ULK1*, *CPTP*, and *NOD2*. In this study, we selected six genes from the nine identified PRDEGs for subsequent analysis. The selection was based on multifactorial considerations, including the stability of the statistical model and biological relevance. The importance of the genes was determined using the random forest algorithm, followed by further screening with LASSO regression to ensure that the selected genes contributed maximally to the model. Additionally, the chosen genes demonstrated potential associations with the pyroptosis pathway and its role in diabetic wound healing. This selection process not only enhanced the robustness of the model but also provided a solid foundation for future extension studies. The results are visualized in Fig. 6D and E, and a forest map of these key genes is presented in Fig. 6F. These genes were used as the key genes in our subsequent studies.

### Construction of a logistic regression model for disease diagnosis via key genes

We developed a diagnostic model for DWH utilizing a multivariate logistic regression model with 6 key genes (*FSTL1*, *PINK1*, *ULK1*, *HDAC3*, *NOD2*, and *CPTP*). The risk score for each sample was calculated by substituting the expression values and weights of these genes from the GSE147890 dataset into the risk score equation. The DWH group was categorized into low- and high-risk groups based on the median risk score.

$$\eqalign{ \>{\rm{Risk}}Score\> = \> & - 29.9355\> * \>FSTL1 + 149.1502\> * \>PINK1 \cr & + 12.0898\> * \>ULK1 + 73.8888\> * \>HDAC3 \cr & + 30.7392\> * \>NOD2 + 39.5365\> * \>CPTP \cr} $$

**Fig. 7:**
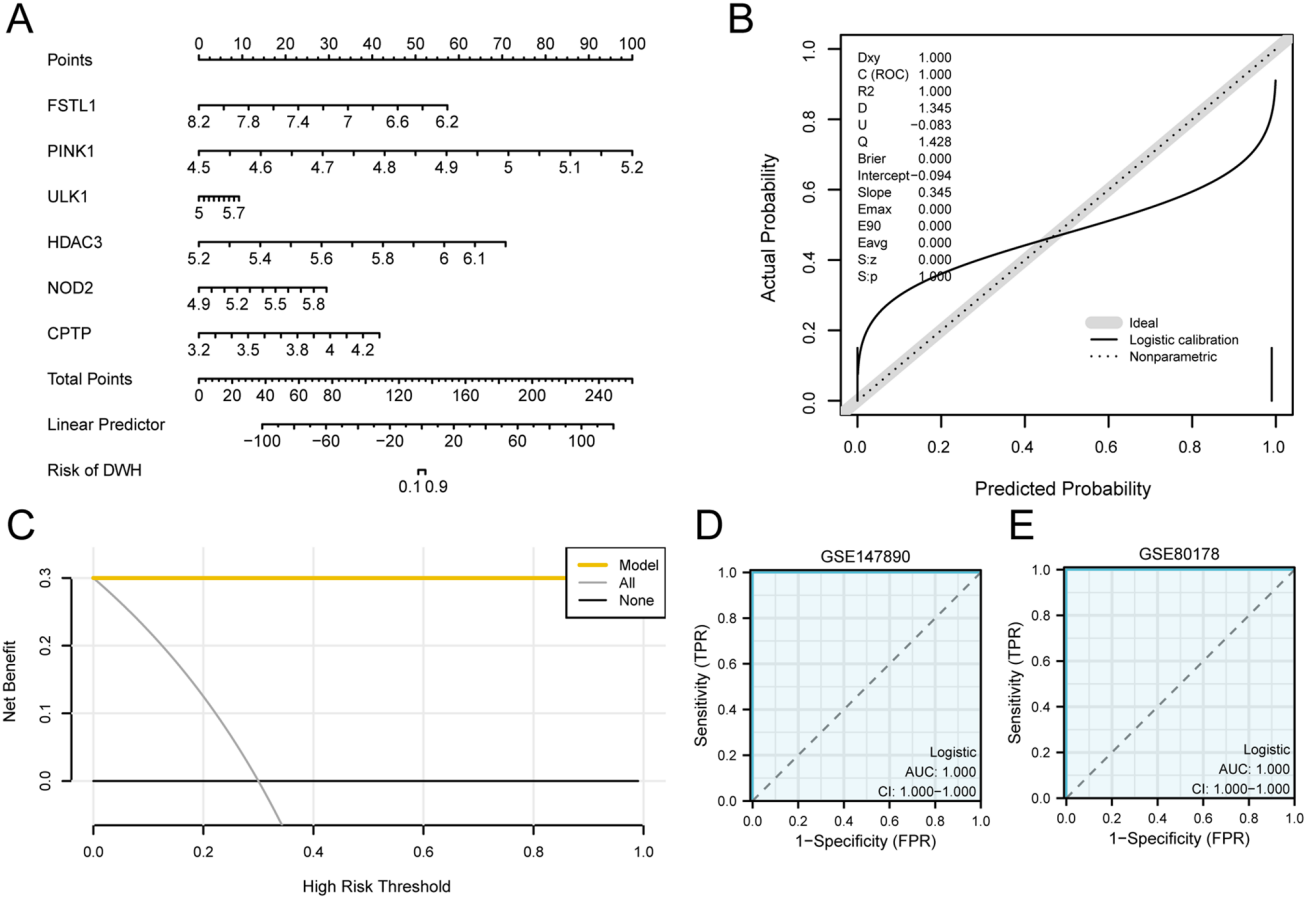
Key genes used to construct the diagnostic logistic regression model (**A**) RF training error: Trees (0–300) vs. error rate. Black line: training error; red line: test error. (**B**) PRDEGs MDG Scatter: MDG on the x-axis shows gene importance for DWH vs. control. Genes sorted by MDG. (**C**) Cross-validation error: gene count on the x-axis and cross-validation error on the y-axis. (**D**) LASSO regression trace: Log lambda on the x-axis and gene regression coefficients on the y-axis. Lines show gene importance. (**E**) LASSO Regression Diagnostic: Bremner’s Bias vs. Log(lambda). Red line: optimal lambda. (**F**) LASSO Key Gene Forest: Summarizes selected genes (FSTL1, PINK1, ULK1, HDAC3, NOD2, CPTP) with coefficients. The coefficient size indicates the diagnostic power for DWH

The nomogram (Fig. 7A) revealed the 6 key links between genes, as shown by the figure showing that *PINK1* expression made the greatest contribution to the multifactor logistic model. To assess the precision and discriminative ability of the multivariate logistic regression model, a calibration curve was generated through calibration analysis. The model’s predictive performance was gauged by examining the alignment between actual probabilities and model-predicted probabilities across various scenarios (Fig. 7B). The calibration curve for the multivariate logistic model revealed that the calibration line (dashed line) closely aligned with the ideal model’s diagonal, suggesting the high accuracy and resolution of the model. 

The diagnostic performance of the diagnostic multivariate logistic model for DWE was subsequently evaluated via DCA on the GSE147890 dataset (Fig. 7C). The findings revealed that the model’s curve remained consistently above those of All and None within a specific range, with the model yielding a greater net benefit, suggesting superior diagnostic performance.

 Finally, the pROC package was used to plot ROC curves for the risk scores from both the GSE147890 and GSE80178 datasets to confirm the diagnostic utility of the multivariate logistic model for DWH (Figs. 7D, E; AUC = 1.000 for both datasets).

### Differential expression verification analysis and functional overlap analysis of key genes

**Fig. 8:**
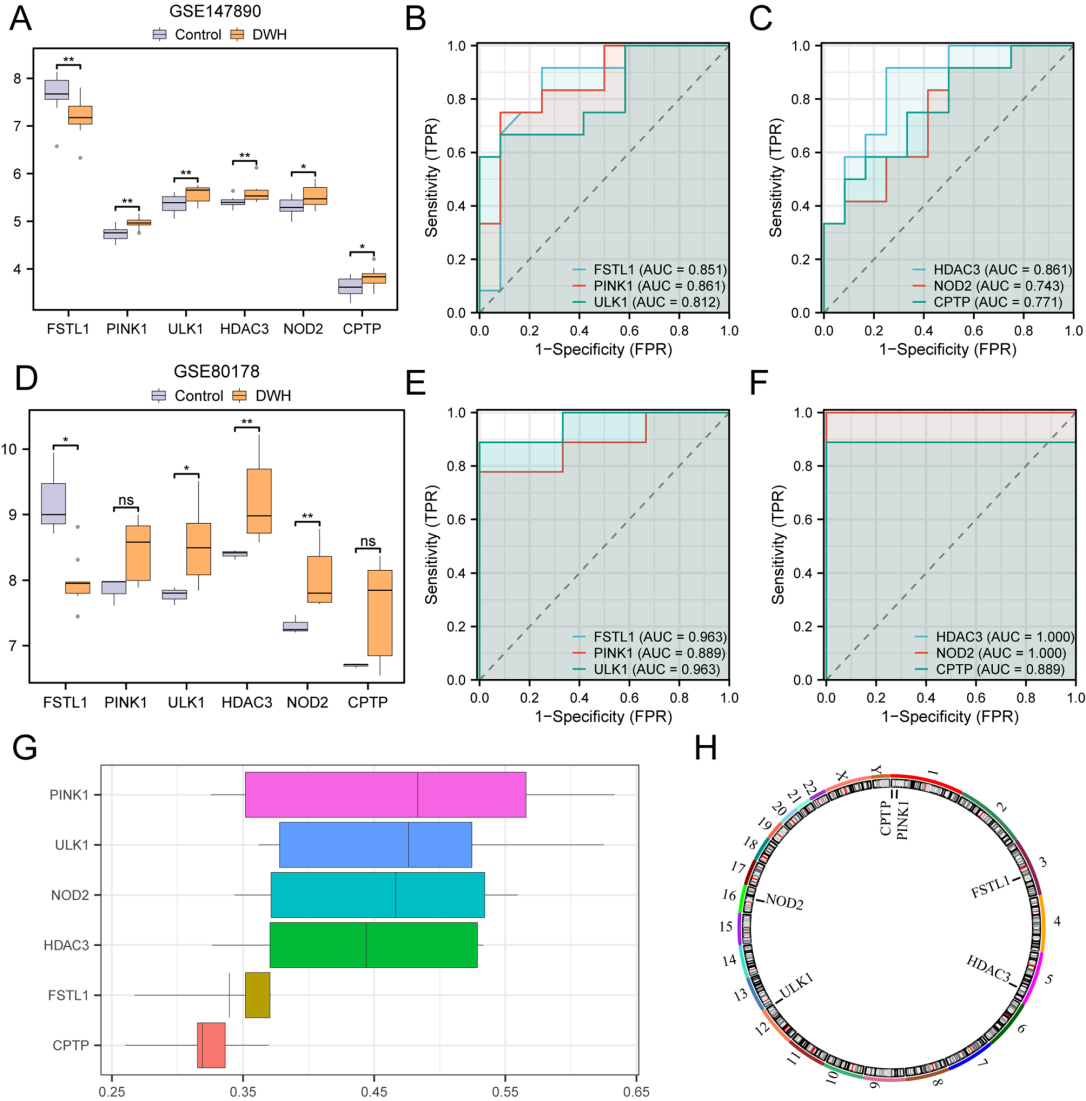
Verification of key gene expression differences **A**. Comparison of key gene expression between the DWH and control groups in GSE147890. **B**-**C**. ROC curves for HDAC3, NOD2 and CPTP in GSE147890. **D**. Comparison of key gene expression between the DWH and control groups in GSE80178. **E**-**F**. ROC curves for FSTL1, PINK1 and ULK1 in the GSE80178 dataset. **G**. Functional similarity analysis of key genes. **H**. Chromosomal localization of key genes. ns: P ≥ 0.05, not significant; *: P < 0.05, significant; **: P < 0.01, highly significant. An AUC > 0.5 indicates a trend toward event occurrence, with an AUC closer to 1 indicating better diagnostic performance. TPR, true positive rate; FPR, false positive rate; Purple: control group; Orange: DWH group

To examine the unique expression profiles of genes (*FSTL1*, *PINK1*, *ULK1*, *HDAC3*, *NOD*2, and *CPTP*) in the GSE147890 dataset, a comparison plot (Fig. 8A) was created to depict the differential expression of these genes between the DWH and control cohorts. The analysis revealed that *FSTL1*, *PINK1*, *ULK1*, and *HDAC3* were significantly differentially expressed between the two groups (*P* < 0.01). ROC curves generated via the pROC package in R revealed that the expression of these genes, including *NOD2* and *CPTP*, had moderate diagnostic accuracy (0.7 < AUC < 0.9) for distinguishing DWH patients from controls (Fig. 8B-C).

The same method was employed to examine the varying expression of key genes (*FSTL1*, *PINK1*, *ULK1*, *HDAC3*, *NOD2*, *CPTP*) in the GSE80178 dataset. A comparison chart (Fig. 8D) was constructed to show the expression levels of the 6 key genes in the GSE80178 DWH group and the control group, and the analysis revealed differences in the expression levels between the high- and low-expression groups. The results revealed differences (Fig. 8D), and two key genes within the GSE80178 dataset presented highly significant differences in gene expression levels between the two groups (*P* value < 0.01): *HDAC3* and *NOD2*. Finally, the R package pROC was used to draw the ROC curve. The ROC curve (Fig. 8E-F) indicated that the expression levels of *FSTL1*, *ULK1*, *HDAC3* and *NOD2* among the key genes exhibited high precision (AUC > 0.9) in distinguishing the DWH group from the control group. *PINK1* and *CPTP* expression levels in the DWH group and control group classification of moderate diagnostic efficacy (0.7 < AUC < 0.9).

The scores of functional similarity (Friends) analysis were used to determine the genes that are pivotal in the biological process of DWH (Fig. 8G). Pivotal genes: In the study of diabetic wound healing, genes were selected through univariate logistic regression analysis (*P* < 0.05) and further verified and selected as key genes via the random forest (RF) model and LASSO regression. These findings indicate that *PINK1* is a key gene in DWH and is nearest to the threshold of significance. (cutoff value = 0.60).

RCircos, an R package, was used to chart the chromosomal locations of the 6 key genes in the human genome, resulting in a chromosome localization map (Fig. 8H). The map indicates the concentrations of key genes on chromosome 1, including *PINK1* and *CPTP*.

### Analysis of immune infiltration of key genes via the SsGSEA method

**Fig. 9:**
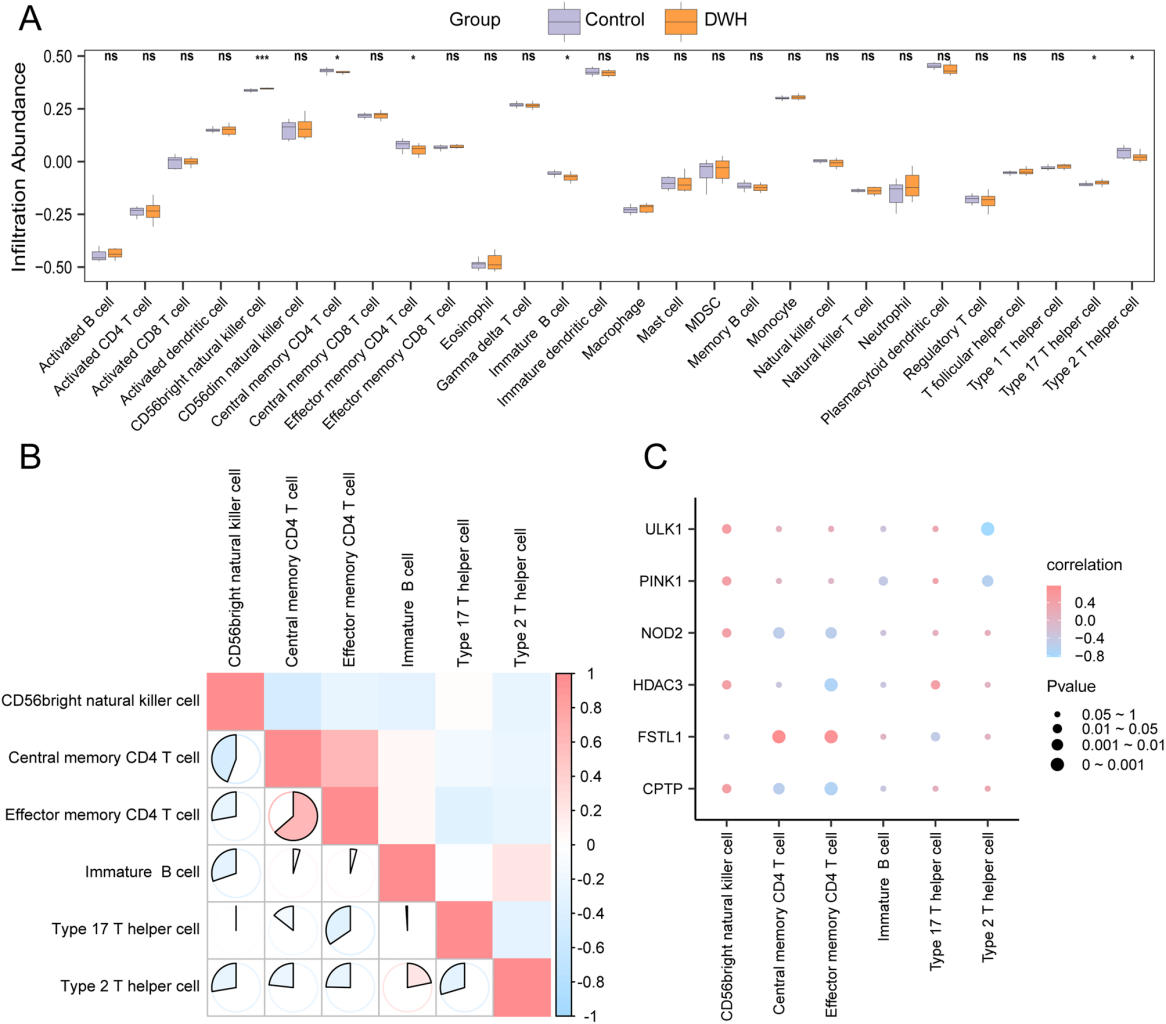
ssGSEA of immune cell infiltration (**A**) Comparison of immune cell infiltration between the DWH and control groups in the GSE147890 dataset. (**B**) Correlation heatmap of immune cell infiltration in GSE147890. (**C**) Bubble plot showing correlations between key gene abundance and immune cells in GSE147890. ns: P ≥ 0.05, not significant; *: P < 0.05, significant; **: P < 0.01, highly significant. r value: Moderate correlation, 0.5–0.8; strong correlation, >0.8. Purple: Control group; Orange: DWH group. Red: positive correlation; blue: negative correlation; color intensity: correlation strength

The ssGSEA algorithm was utilized to quantify the immune infiltration levels of 28 cell types via expression data from the GSE147890 dataset. Through group comparison plots, 6 immune cell types with significant differences (*P* < 0.05) between the DWH and control groups were identified, as depicted in Fig. 9A. A correlation heatmap (Fig. 9B) revealed the relationships among the infiltrating immune cells, predominantly negative correlations. The correlation bubble plot (Fig. 9C) revealed that *FSTL1* had the strongest positive correlation with central memory CD4 + T cells (*r* = 0.78, *P* < 0.05), whereas *ULK1* had the strongest negative correlation with Th2 cells (*r* = -0.82, *P* < 0.05).

### GSEA of the high- and low-logistic risk score groups

**Fig. 10:**
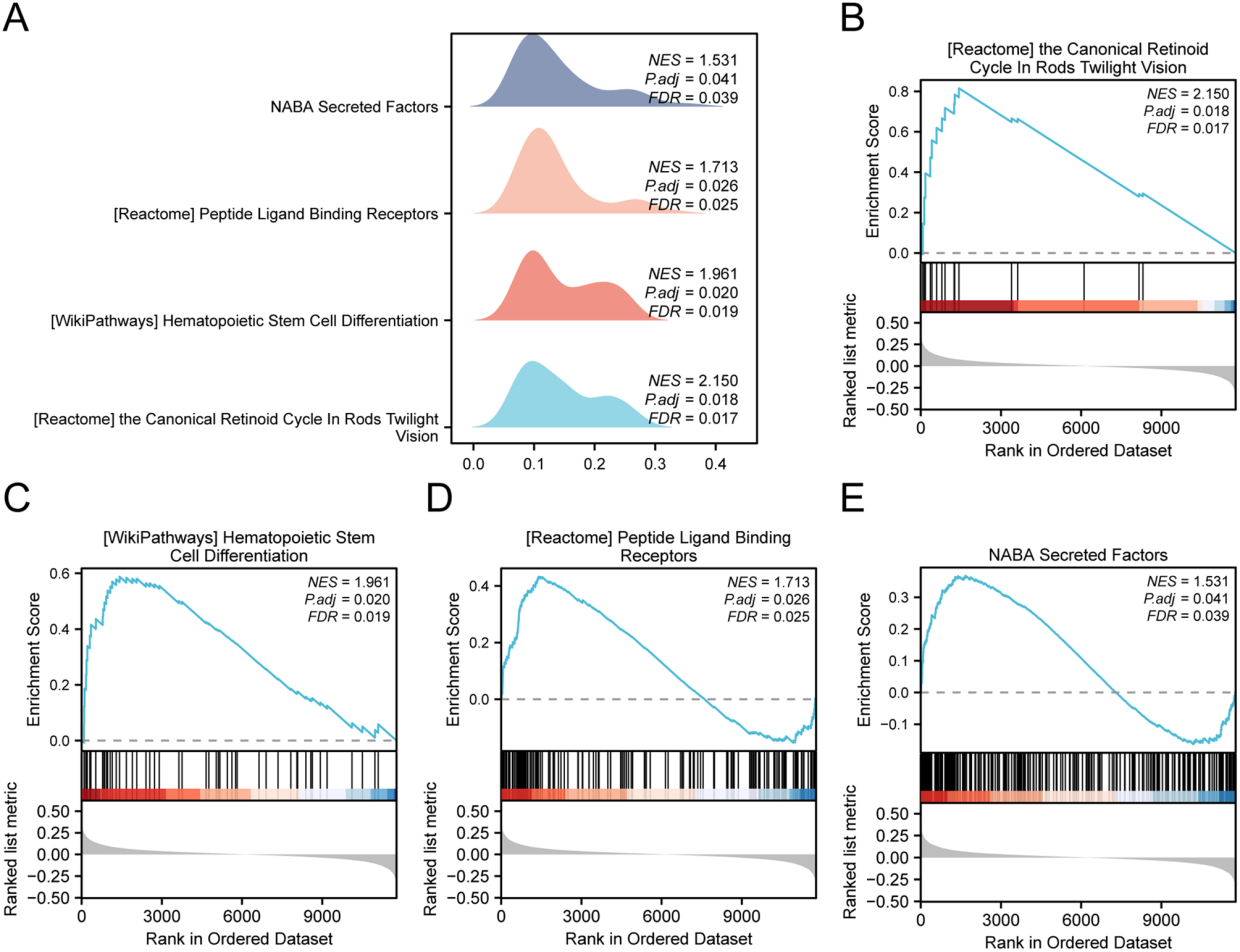
diabetic wound healing GSEA of high and low risk group **A**. Data set GSE147890 GSEA mountains diagram shows four biological functions. **B**-**E**. GSEA revealed that all genes were significantly enriched in the Canonical Retinoid Cycle in Rods Twilight Vision (**B**). Hematopoietic Stem Cell Differentiation (**C**). Peptide Ligand Binding Receptors (**D**). NABA Secreted Factors (**E**). GSEA selection criteria for adj. (P < 0.05) and FDR value (q value) < 0.25, P value correction methods for Benjamini - Hochberg (BH)

GSEA was utilized to assess how the expression of all 12,548 genes in the GSE147890 dataset impacts the distinction between high- and low-risk score groups for DWH. The analysis aimed to determine the BPs, CCs, and MFs associated with DWH in the high- versus low-risk score groups (Supplementary Fig. 10A). For specific results, see Supplementary Table S9. According to the results of the dataset, all the genes in GSE147890 were significantly enriched in the Canonical Retinoid Cycle Rods in Twilight Vision (Supplementary Fig. 10B), Hematopoietic Stem Cell Differentiation (Supplementary Fig. 10C), Peptide Ligand Binding Receptors (Supplementary Fig. 10D), NABA Secreted Factors (Supplementary Fig. 10E), and other biologically related functions and signaling pathways (Fig. 10).

### Analysis of mRNA‒miRNA and mRNA‒transcription factor interactions

**Fig. 11:**
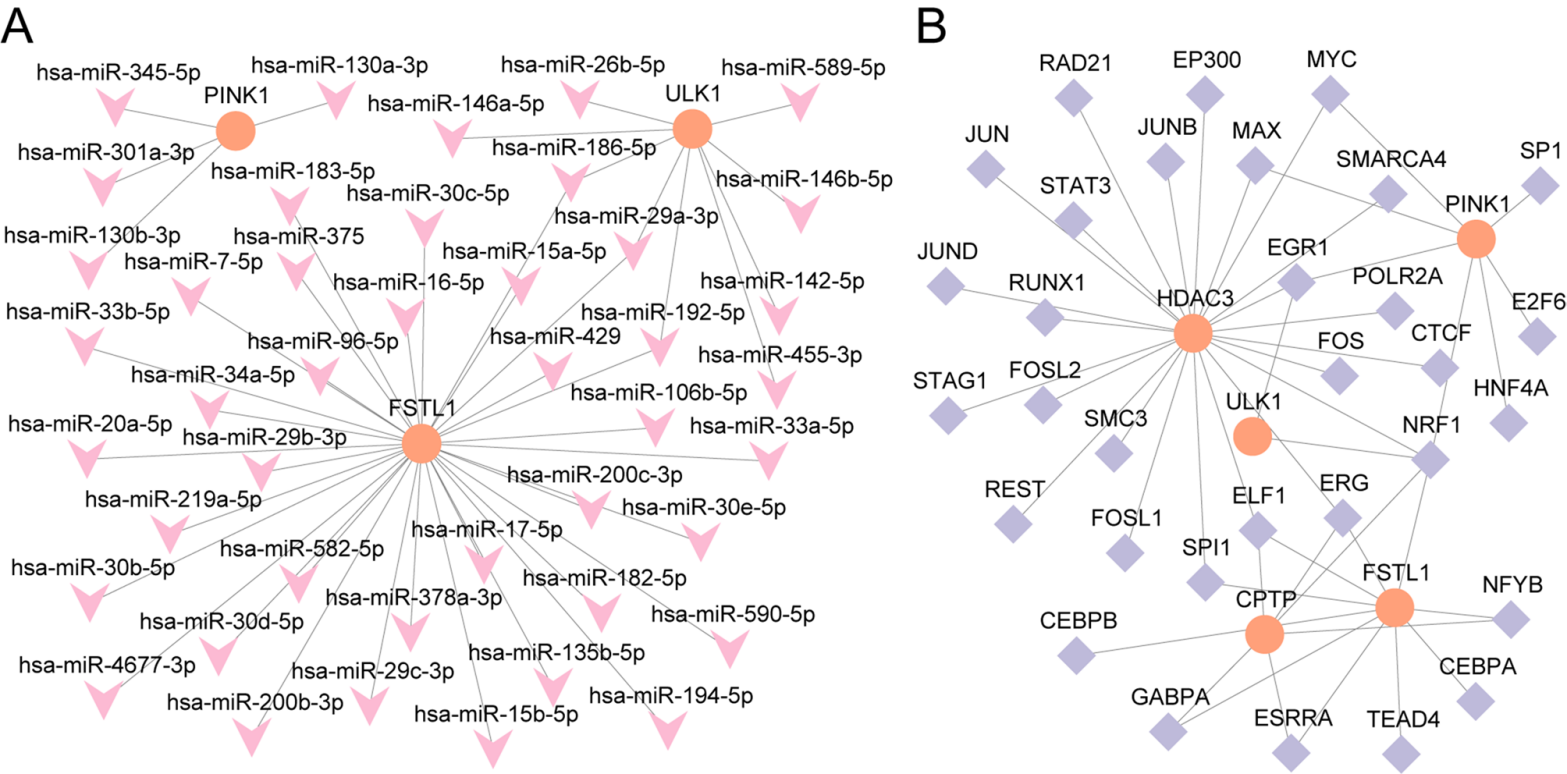
Interaction network analysis of key genes. (**A**) mRNA-miRNA interaction network of key genes. (**B**) mRNA-TF interaction network of key genes. TF, Transcription Factor. Orange is mRNA, purple is TF, and pink is miRNA

ENCORI was utilized to identify miRNAs that interact with key genes (*FSTL1*, *PINK1*, *ULK1*, *HDAC3*, *NOD2*, and *CPTP*), resulting in an mRNA‒miRNA interaction network visualized via Cytoscape (Supplementary Fig. 11A). This network included 3 key genes and 43 miRNAs, as detailed in Supplementary Table S10. Additionally, ChIPBase was used to identify transcription factors (TFs) associated with the key genes, leading to the construction of an mRNA‒TF interaction network, which was also visualized via Cytoscape (Supplementary Fig. 11B). This network comprises 5 key genes (*FSTL1*, *PINK1*, *ULK1*, *HDAC3*, and *CPTP*) and 32 TFs, with specific details provided in Supplementary Table S11. The mRNA‒miRNA and mRNA‒TF interaction networks are collectively visualized in Fig. 11.

### Differential expression of the key genes

**Fig. 12:**
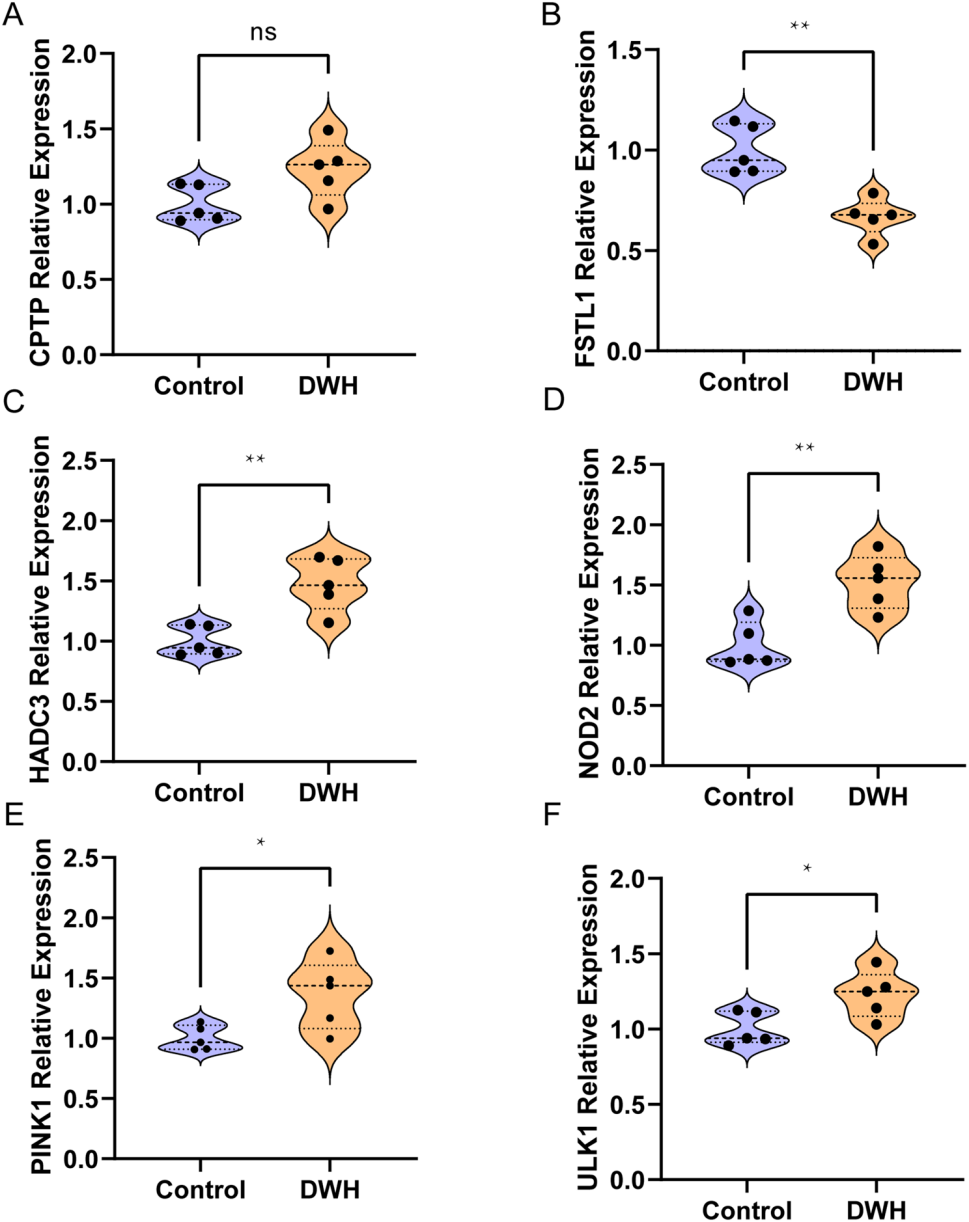
Relative expression of the 6 key genes. **A**–**F**. Differences in the expression of the key genes between the DWH and control groups. CPTP relative expression (**A**); FSTL1 relative expression (**B**); HADC3 relative expression (**C**); NOD2 relative expression (**D**); PINK1 relative expression (**E**). ULK1 relative expression (**F**). ns > 0.05; *P < 0.05; ** P < 0.01

To corroborate our findings, quantitative PCR (qPCR) was employed to measure the mRNA expression of the 6 key genes in five pairs of skin biopsies from diabetic wounds (DWH group) and acute wounds (control group). The qPCR results revealed that genes such as *PINK1*, *HDAC3*, *ULK1*, and *NOD2* were significantly overexpressed in the DWH group compared with the control group, whereas the relative expression levels of *FSTL1* were significantly decreased. In contrast, the relative expression of *CPTP* did not significantly differ between the two groups (Fig. 12).

## Discussion

Diabetic wounds, particularly foot ulcers, pose a significant health challenge and often result in prolonged hospitalization, increased healthcare costs, and increased risks of infection and amputation [34]. DFUs exhibit clinical heterogeneity due to biological, genetic, and environmental factors, complicating wound healing. Pathogenesis involves multiple pathways, with local hyperglycemia, oxidative stress, and chronic inflammation playing key roles. Persistent inflammation activation is the primary cause of chronic nonhealing diabetic wounds and significantly contributes to DFUs, gangrene, and amputation [35], and pyroptosis is also involved. Given the increasing global prevalence of diabetes, understanding the influence of pyroptosis on sustained inflammation and its molecular mechanisms that impede diabetic wound healing is crucial for the development of effective targeted therapies, improving healing in patients with diabetic foot ulcers, and reducing the incidence of amputation [7].

Pyroptosis, a form of inflammatory programmed cell death, is associated with various diseases, including diabetes. Appropriate inflammation is a critical component of the physiological wound healing phase, particularly within the diabetic wound milieu, where it is essential for the removal of cellular debris and potential pathogens, including bacteria [36]. In diabetic wounds, the activation of inflammasomes is especially pivotal due to tissue damage and impaired repair functions resulting from hyperglycemia. These intracellular multiprotein complexes are capable of recognizing pathogen-associated molecular patterns (PAMPs) or damage-associated molecular patterns (DAMPs) and are activated in response to tissue injury. The activation of inflammasomes leads to the cleavage of the caspase-1 precursor into its active form, triggering pyroptosis, which is instrumental in controlling inflammation and preventing the spread of pathogens [37]. During the wound healing process, inflammatory cells undergo orderly pyroptotic cell death, which is crucial for regulating inflammation and promoting the clearance of pathogens and damaged cells, thereby facilitating healing. However, the unique characteristics of diabetic wounds may impair leukocyte infiltration and clearance functions; if not effectively removed via pyroptosis or apoptosis, the healing process may be delayed or suppressed, potentially leading to a persistent pro-inflammatory state and the development of non-healing chronic wounds [38].

Therefore, precise regulation of the balance of pyroptosis is essential to avoid chronic inflammation and to promote effective healing of diabetic wounds. Bioinformatics analysis, with its systematic approach, large data volume, and reliable results, has emerged as a valuable tool for exploring the intricate molecular mechanisms underlying the evolution and progression of DFU disease [39].This study employs bioinformatics methods to analyze the relationships between differentially expressed genes (DEGs) and pyroptosis, aiming to identify key genes and pathways associated with diabetic wound healing, construct diagnostic models, and validate the transcription of key genes through in vitro experiments.

In the context of DWH, a total of 6 key PRDEGs (*FSTL1*, *PINK1*, *HDAC3*, *ULK1*, *CPTP*, and *NOD2*) were identified as key genes with significantly differential expression. *HDAC3* (histone deacetylase 3) is an important deacetylase that plays a pivotal role in regulating gene expression through chromatin remodeling [40]. It is engaged in numerous cellular processes, including inflammation, cell proliferation, and differentiation, and it plays a role in the regulation of various signaling pathways, including NF-κB activity, thereby influencing pyroptosis [41]. Previous research has shown that the NLRP3 inflammasome is a multiprotein complex that significantly influences the activation of inflammation in the pathogenesis of innate immunity and inflammation-related diseases, such as cardiovascular diseases, neurodegenerative diseases, cancer, type 2 diabetes, and diabetic wounds [42]. *HDAC3* serves as a critical factor in the activation of the NLRP3 inflammasome. Research has indicated that *HDAC3* knockdown significantly suppresses the activities of the NLRP3 inflammasome and pyroptosis, thereby mitigating inflammation and pyroptosis and enhancing disease prognosis [43, 44]. The strong association of *HDAC3* with critical signaling pathways, such as the NF-κB pathway, further underscores its role in modulating inflammatory responses during wound healing. In this study, *HDAC3* expression was significantly different between the two independent databases, a finding further corroborated by qRT‒PCR analysis, confirming the precision of *HDAC3* as a key gene. We hypothesize that high expression of *HDAC3* may contribute to delayed wound healing in DFUs.

The involvement of *PINK1* in mitochondrial autophagy suggests that it might promote or accompany the activation of pyroptosis [45] aiding in the alleviation of oxidative stress and the maintenance of cellular homeostasis, which are crucial for effective wound healing [46]. Follistatin-like 1 (FSTL1) is a secreted glycoprotein that has been implicated in various biological processes, including inflammation, fibrosis, and tissue repair. Previous studies have demonstrated that *FSTL1* acts as a crucial mediator of inflammation in the development of certain inflammatory diseases, promoting synoviocyte proliferation through the activation of the NF-κB pathway [47]. Unlike other key genes, *Unc-51* Like Autophagy Activating Kinase 1 (ULK1) may negatively regulate inflammasome activation [48]. Studies have shown that *ULK1* can inhibit the activity of the NLRP3 inflammasome in the pyroptosis pathway [49] potentially aiding in mitigating the harmful effects of hyperglycemia-induced oxidative stress and inflammation in diabetic wounds. Nucleotide-binding oligomerization domain-containing protein 2 (NOD2) is a pattern recognition receptor that, like *NLRP3*, belongs to the NOD-like receptor (NLR) family. Activated *NOD2* can recruit Receptor Interacting Protein 2 (RIP2) and influence the activity of immune cells and the secretion of proinflammatory cytokines through the NF-κB and MAPK pathways [50] thereby affecting the wound healing process.

To further validate our findings, we employed quantitative PCR (qPCR) to assess the mRNA expression levels of six key genes in skin biopsy samples obtained from five pairs each of diabetic wound (DWH group) and acute wound (control group) tissues. The results demonstrated that in the diabetic wound group, the mRNA expression levels of PINK1, HDAC3, ULK1, and NOD2 were elevated, whereas the expression of FSTL1 was significantly decreased compared to the acute wound (control) group. No significant difference in CPTP expression was observed between the two groups. Due to the challenges associated with clinical sample collection and ethical considerations, the sample size of this validation cohort was limited. Nevertheless, the qPCR results were highly consistent with our bioinformatics predictions, supporting the involvement of these key genes in diabetic wound healing. We acknowledge that the limited sample size may affect the generalizability of the results; therefore, future studies will aim to expand the sample size and further validate and extend these findings in larger and more diverse populations. Future research should focus on cellular changes in diabetic wounds under hyperglycemic conditions to investigate how alterations in gene expression influence pyroptosis pathways. This comprehensive strategy will help validate the role and mechanisms of pyroptosis genes in diabetic wound healing.

The analysis of the 9 PRDEGs highlights their significant involvement in various key biological processes and pathways, which are crucial for deciphering the molecular mechanisms underlying DWH. The IKK/NF-κB pathway is essential for regulating immune responses and inflammation. Disrupted regulation of this pathway is linked to persistent inflammatory conditions involving impaired wound healing in individuals with diabetes [51]. Moreover, another critical signaling pathway, the JNK cascade, also plays a key role in cellular stress responses, apoptosis, and inflammation, and its dysregulated activation has been associated with delayed wound healing and increased inflammation in diabetic patients [52]. Disruptions in pattern recognition receptor signaling pathways, including Toll-like receptors (TLRs) and NOD-like receptors (NLRs), can result in impaired immune responses and chronic inflammation, thereby exacerbating poor wound healing in diabetes [53].

Mitophagy, the selective degradation of mitochondria by autophagy, is crucial for maintaining cellular homeostasis and energy production. Dysregulation of mitophagy has been linked to increased oxidative stress and impaired wound healing in diabetic conditions. The enrichment of PRDEGs in the influenza A pathway suggests a potential overlap between viral infection responses and wound healing, reflecting the complex interplay between immune responses and tissue repair [54]. However, given that this pathway is commonly observed in various inflammatory diseases, it is more likely a shared inflammatory signal resulting from database annotations rather than a direct biological mechanism linking DWH to influenza A infection. Therefore, we believe this indicates a general overlap between inflammatory and immune pathways, and its interpretation should be conducted with caution, considering the specific biological context. The NOD-like receptor signaling pathway participates in the control of inflammation and programmed cell death. Its dysregulation has been associated with chronic inflammatory diseases and impaired wound healing [55]. Overall, the results underscore the importance of PRDEGs at DWH and provide a foundation for future research aimed at developing targeted interventions and diagnostic techniques for DWH.

In this research, we performed an extensive analysis of immunocyte infiltration to elucidate the involvement of various immunocytes in DWH. The analysis revealed substantial differences in the abundances of central memory and effector memory CD4 + T cells, immature B cells, Th17 and Th2 cells, and CD56bright NK cells between the two groups. Central memory CD4 + T cells and effector memory CD4 + T cells can mount rapid and robust responses upon re-exposure to antigens [56]. Immature B cells play a pivotal role in humoral immunity and indicate ongoing immune responses [57]. Th17 cells, known for their production of cytokines such as IL-17, can exacerbate inflammatory and autoimmune responses [58]. Concurrently, Th2 cells, which are involved in anti-inflammatory responses, tissue repair, and the production of cytokines such as IL-4 and IL-10 [59], along with CD56bright NK cells that regulate immune responses by secreting high levels of cytokines [60] both show changes in their infiltration levels. Overall, these findings highlight the complex interplay of diverse immunocyte types in the pathophysiology of DWH.

The interaction analysis between these immune cells and the 6 key genes revealed significant correlations. For example, *FSTL1* was strongly positively correlated with central memory CD4 + T cells (*r* = 0.78), suggesting that *FSTL1* may influence adaptive immune responses in the DWH. Conversely, *ULK1* exhibited a strong negative correlation with Th2 cells (*r*=-0.82), indicating its potential role in regulating anti-inflammatory responses. These findings imply that the identified PRGs are not only involved in the pyroptosis pathway but also crucial for modulating the infiltration and activity of immune cells in diabetic wounds. This underscores the potential for developing novel therapeutic strategies targeting specific immune cells and pathways to enhance wound healing in diabetic patients, which requires validation in a broader patient population in subsequent studies.

This study primarily relied on data from two public databases, GSE147890 and GSE80178. The relatively small sample sizes and diverse origins of these datasets may limit the generalizability of our findings. Although batch effects were effectively corrected using established methods, and box plots indicated that these effects were largely mitigated, the potential for residual bias remains. Such bias could stem from biological variability, technical errors, and the inherent limitations of the statistical methods employed. These factors might influence the accuracy of identifying differentially expressed genes, thereby affecting the reliability of our results. Consequently, interpretation of these findings should be approached with caution.

To evaluate the diagnostic performance of the multivariate logistic regression model we developed for DWH, we employed the pROC package to generate ROC curves for the model’s risk scores using the GSE147890 and GSE80178 datasets (Figs. 7D, E). The results demonstrated that the model achieved an exceptionally high AUC value (AUC = 1.000) in both datasets. Notably, such a high AUC might be attributable to the limited sample sizes and relatively homogeneous origins of the datasets, which again highlights the potential impact of the previously mentioned small sample sizes and data diversity. While this preliminary result suggests promising diagnostic potential for the model, its generalizability remains to be confirmed in larger-scale and more heterogeneous populations due to the lack of an independent external validation cohort.

The findings of this study are largely based on bioinformatics analysis, with experimental validation performed for only a limited number of genes. Comprehensive experimental validation, including in vitro and in vivo studies, is necessary to confirm the specific roles of these genes in the pathophysiology of the disease. Furthermore, the absence of an external validation dataset means that our model and findings may exhibit different performance in other independent samples or distinct populations. By acknowledging these limitations, we hope that the preliminary insights derived from this study can lay a solid foundation for future, more comprehensive, and in-depth investigations. Future plans include collecting additional external clinical samples and implementing more rigorous cross-validation methods to mitigate the risk of overfitting and enhance the robustness of the model, thereby contributing to advancements in the field of diabetic foot ulcer (DFU) treatment.

Overall, this research effectively pinpointed DEGs linked to pyroptosis in DWH and constructed a diagnostic model with promising accuracy. Our findings highlight significant biological pathways and immunocyte interactions relevant to the wound healing process in diabetic patients. The constructed mRNA‒miRNA and mRNA‒TF interaction networks provide a deeper understanding of the regulatory mechanisms at play. Future research should focus on validating these results through clinical trials and experimental studies to increase the stability and practicality of our diagnostic model, potentially leading to improved therapeutic strategies for DWH.

Overall, this study effectively identified DEGs associated with pyroptosis in DWH and constructed a diagnostic model demonstrating promising accuracy. This study systematically screened for DEGs related to pyroptosis and, based on multi-omics analysis and basic experimental validation, preliminarily revealed their potential roles in DWH. Our findings highlighted significant biological pathways and immune cell interactions relevant to the wound healing process in diabetic patients. Regarding the molecular mechanisms by which the identified key genes regulate pyroptosis and influence DWH, we provided a preliminary discussion based on the existing data; however, further in-depth experimental research is required for elucidation. The constructed mRNA-miRNA and mRNA-TF interaction networks offered deeper insights into the underlying regulatory mechanisms. Concerning the clinical application and therapeutic implications of diagnostic biomarkers, the current results provide a foundational reference for related mechanisms and individualized management, although validation in larger sample sizes and multicenter cohorts is still needed. Future research should focus on validating these findings through clinical trials and experimental studies to enhance the stability and practicality of our diagnostic model, potentially leading to improved therapeutic strategies for DWH. In summary, our research has laid a preliminary foundation for subsequent mechanistic studies and clinical translation.

## Supplementary Information

Below is the link to the electronic supplementary material.

Supplementary Material 1

Supplementary Material 2

Supplementary Material 3

Supplementary Material 4

Supplementary Material 5

Supplementary Material 6

Supplementary Material 7

Supplementary Material 8

Supplementary Material 9

Supplementary Material 10

Supplementary Material 11

## Data Availability

The datasets (GSE147890 and GSE80178) analyzed in this study were extracted from the Gene Expression Omnibus database (GEO, https://www.ncbi.nlm.nih.gov/geo/).
