# Supplementary material for: Identifying pyroptosis-related genes as novel therapeutic targets in diabetic foot ulceration
Source: Diabetol Metab Syndr. 2025 Aug 1;17:306. doi: 10.1186/s13098-025-01880-9 (PMC12315281; doi:10.1186/s13098-025-01880-9)
Supplement: Supplementary file 5 — Supplementary Material 5 [file 13098_2025_1880_MOESM5_ESM.docx]

### **Supplementary Table S5 Primer used for qRT-PCR**.

| Gene | Primer (5’→ 3’) |
| --- | --- |
| *FSTL1* | F: AATGGCAAGACCTACCTCAACC |
|  | R: GTGCCCATCATAATCAACCTGG |
| *PINK1* | F: GGAGGAGTATCTGATAGGGCAG |
|  | R: AACCCGGTGCTCTTTGTCAC |
| *ULK1* | F: TGCCCCCGGTTGAATGTTCT |
|  | R:ACACCGCCCAACTTCCAC |
| *HDAC3* | F: GACATCGCTGCTGGTAGAAGAGG |
|  | R:GGGTGCTGACATCTGGATGAAGTG |
| *NOD2* | F: CCGCAAGCACTTCCACTCCATC |
|  | R:AGCCGCTCCTCCTGCATCTC |
| *CPTP* | F: TGGTCAGTTTCAAGCAGTGTCTCG |
|  | R:GAAGATGGTGCCCAGGCTGTTC |
| *GAPDH* | F: GTGGACCTGACCTGCCGTCTAG |
|  | R: GAGTGGGTGTCGCTGTTGAAGTC |
