# Supplementary material for: Identifying pyroptosis-related genes as novel therapeutic targets in diabetic foot ulceration
Source: Diabetol Metab Syndr. 2025 Aug 1;17:306. doi: 10.1186/s13098-025-01880-9 (PMC12315281; doi:10.1186/s13098-025-01880-9)
Supplement: Supplementary file 6 — Supplementary Material 6 [file 13098_2025_1880_MOESM6_ESM.docx]

### Supplementary Table S6 Results of GO and KEGG Enrichment Analysis for PRDEGs

| ONTOLOGY | ID | Description | GeneRatio | BgRatio | pvalue | p.adjust | qvalue |
| --- | --- | --- | --- | --- | --- | --- | --- |
| BP | GO:0043122 | regulation of I-kappaB kinase/NF-kappaB signaling | 5/9 | 254/18800 | 5.218E-08 | 3.9696E-05 | 1.5008E-05 |
| BP | GO:0007249 | I-kappaB kinase/NF-kappaB signaling | 5/9 | 288/18800 | 9.7654E-08 | 3.9696E-05 | 1.5008E-05 |
| BP | GO:0046328 | regulation of JNK cascade | 4/9 | 141/18800 | 3.7104E-07 | 0.00010055 | 3.8016E-05 |
| BP | GO:0007254 | JNK cascade | 4/9 | 175/18800 | 8.8143E-07 | 0.00012218 | 4.6194E-05 |
| BP | GO:0002221 | pattern recognition receptor signaling pathway | 4/9 | 176/18800 | 9.0173E-07 | 0.00012218 | 4.6194E-05 |
| CC | GO:0031968 | organelle outer membrane | 4/9 | 232/19594 | 2.3034E-06 | 6.0778E-05 | 3.2616E-05 |
| CC | GO:0019867 | outer membrane | 4/9 | 234/19594 | 2.3834E-06 | 6.0778E-05 | 3.2616E-05 |
| CC | GO:0005741 | mitochondrial outer membrane | 3/9 | 205/19594 | 9.0501E-05 | 0.00153851 | 0.00082562 |
| CC | GO:0005774 | vacuolar membrane | 2/9 | 449/19594 | 0.01695412 | 0.06651233 | 0.03569289 |
| CC | GO:0005667 | transcription regulator complex | 2/9 | 483/19594 | 0.01946324 | 0.07090182 | 0.03804845 |
| MF | GO:0033218 | amide binding | 3/9 | 402/18410 | 0.00078709 | 0.02676095 | 0.00869938 |
| MF | GO:0051059 | NF-kappaB binding | 2/9 | 31/18410 | 9.8064E-05 | 0.00666832 | 0.00216772 |
| MF | GO:0001221 | transcription coregulator binding | 2/9 | 108/18410 | 0.00119495 | 0.02708546 | 0.00880487 |
| MF | GO:0042826 | histone deacetylase binding | 2/9 | 126/18410 | 0.0016212 | 0.02756046 | 0.00895928 |
| MF | GO:0005539 | glycosaminoglycan binding | 2/9 | 234/18410 | 0.00546032 | 0.0315942 | 0.01027056 |
| KEGG | hsa04137 | Mitophagy - animal | 3/7 | 72/8164 | 2.2447E-05 | 0.00190801 | 0.0014886 |
| KEGG | hsa05164 | Influenza A | 3/7 | 171/8164 | 0.00029707 | 0.01044872 | 0.00815194 |
| KEGG | hsa04621 | NOD-like receptor signaling pathway | 3/7 | 184/8164 | 0.00036878 | 0.01044872 | 0.00815194 |
| KEGG | hsa05022 | Pathways of neurodegeneration - multiple diseases | 3/7 | 476/8164 | 0.00577755 | 0.05456574 | 0.04257141 |
| KEGG | hsa05321 | Inflammatory bowel disease | 2/7 | 65/8164 | 0.00127753 | 0.02516419 | 0.01963274 |

GO，Gene Ontology；BP，Biological Process；CC，Cellular Component；MF，Molecular Function；KEGG，Kyoto Encyclopedia of Genes and Genomes；PRDEGs，Pyroptosis-Related Differentially Expressed Genes。
