# Supplementary material for: Identifying pyroptosis-related genes as novel therapeutic targets in diabetic foot ulceration
Source: Diabetol Metab Syndr. 2025 Aug 1;17:306. doi: 10.1186/s13098-025-01880-9 (PMC12315281; doi:10.1186/s13098-025-01880-9)
Supplement: Supplementary file 7 — Supplementary Material 7 [file 13098_2025_1880_MOESM7_ESM.docx]

### Supplementary Table S7 Results of GSEA for GSE147890

| ID | setSize | enrichmentScore | NES | pvalue | p.adjust | qvalue |
| --- | --- | --- | --- | --- | --- | --- |
| REACTOME_TNFR1_INDUCED_PROAPOPTOTIC_SIGNALING | 13 | 0.70013557 | 1.85341015 | 0.00214701 | 0.04998574 | 0.04476138 |
| WP_CORTICOTROPINRELEASING_HORMONE_SIGNALING_PATHWAY | 88 | 0.45173398 | 1.86898943 | 8.8666E-05 | 0.00401171 | 0.00359242 |
| WP_GENES_RELATED_TO_PRIMARY_CILIUM_DEVELOPMENT_BASED_ON_CRISPR | 62 | -0.51333551 | -1.79363218 | 0.00167126 | 0.0414586 | 0.03712547 |
| REACTOME_DISEASES_ASSOCIATED_WITH_GLYCOSAMINOGLYCAN_METABOLISM | 35 | -0.59201536 | -1.83467991 | 0.00069576 | 0.02085528 | 0.01867555 |

GSEA，Gene Set Enrichment Analysis。
