# Supplementary material for: Identifying pyroptosis-related genes as novel therapeutic targets in diabetic foot ulceration
Source: Diabetol Metab Syndr. 2025 Aug 1;17:306. doi: 10.1186/s13098-025-01880-9 (PMC12315281; doi:10.1186/s13098-025-01880-9)
Supplement: Supplementary file 9 — Supplementary Material 9 [file 13098_2025_1880_MOESM9_ESM.docx]

### Supplementary Table S9 Results of GSEA（Low/High）for GSE147890

| ID | setSize | enrichmentScore | NES | pvalue | p.adjust | qvalue |
| --- | --- | --- | --- | --- | --- | --- |
| REACTOME_THE_CANONICAL_RETINOID_CYCLE_IN_RODS_TWILIGHT_VISION | 16 | 0.81519229 | 2.15009589 | 1.8624E-05 | 0.01797181 | 0.01716635 |
| WP_HEMATOPOIETIC_STEM_CELL_DIFFERENTIATION | 51 | 0.58790292 | 1.96100605 | 4.1501E-05 | 0.01975768 | 0.01887218 |
| REACTOME_PEPTIDE_LIGAND_BINDING_RECEPTORS | 164 | 0.43236766 | 1.71292917 | 7.529E-05 | 0.02579236 | 0.02463639 |
| NABA_SECRETED_FACTORS | 263 | 0.36731541 | 1.53082394 | 0.00017106 | 0.04101968 | 0.03918126 |

GSEA，Gene Set Enrichment Analysis。
