# Supplementary material for: Identifying pyroptosis-related genes as novel therapeutic targets in diabetic foot ulceration
Source: Diabetol Metab Syndr. 2025 Aug 1;17:306. doi: 10.1186/s13098-025-01880-9 (PMC12315281; doi:10.1186/s13098-025-01880-9)
Supplement: Supplementary file 10 — Supplementary Material 10 [file 13098_2025_1880_MOESM10_ESM.docx]

### Supplementary Table S10 mRNA-miRNA Interaction Network Nodes

| mRNA | miRNA |
| --- | --- |
| FSTL1 | hsa-miR-15a-5p |
| FSTL1 | hsa-miR-16-5p |
| FSTL1 | hsa-miR-17-5p |
| FSTL1 | hsa-miR-20a-5p |
| FSTL1 | hsa-miR-29a-3p |
| FSTL1 | hsa-miR-33a-5p |
| FSTL1 | hsa-miR-96-5p |
| FSTL1 | hsa-miR-29b-3p |
| FSTL1 | hsa-miR-192-5p |
| FSTL1 | hsa-miR-30c-5p |
| FSTL1 | hsa-miR-30d-5p |
| FSTL1 | hsa-miR-7-5p |
| FSTL1 | hsa-miR-34a-5p |
| FSTL1 | hsa-miR-182-5p |
| FSTL1 | hsa-miR-183-5p |
| FSTL1 | hsa-miR-219a-5p |
| FSTL1 | hsa-miR-200b-3p |
| FSTL1 | hsa-miR-15b-5p |
| FSTL1 | hsa-miR-30b-5p |
| FSTL1 | hsa-miR-186-5p |
| FSTL1 | hsa-miR-194-5p |
| FSTL1 | hsa-miR-200c-3p |
| FSTL1 | hsa-miR-106b-5p |
| FSTL1 | hsa-miR-29c-3p |
| FSTL1 | hsa-miR-30e-5p |
| FSTL1 | hsa-miR-375 |
| FSTL1 | hsa-miR-378a-3p |
| FSTL1 | hsa-miR-135b-5p |
| FSTL1 | hsa-miR-429 |
| FSTL1 | hsa-miR-582-5p |
| FSTL1 | hsa-miR-590-5p |
| FSTL1 | hsa-miR-33b-5p |
| FSTL1 | hsa-miR-4677-3p |
| PINK1 | hsa-miR-130a-3p |
| PINK1 | hsa-miR-301a-3p |
| PINK1 | hsa-miR-130b-3p |
| PINK1 | hsa-miR-345-5p |
| ULK1 | hsa-miR-26b-5p |
| ULK1 | hsa-miR-29a-3p |
| ULK1 | hsa-miR-192-5p |
| ULK1 | hsa-miR-142-5p |
| ULK1 | hsa-miR-146a-5p |
| ULK1 | hsa-miR-186-5p |
| ULK1 | hsa-miR-146b-5p |
| ULK1 | hsa-miR-455-3p |
| ULK1 | hsa-miR-589-5p |
