# Supplementary material for: Identifying pyroptosis-related genes as novel therapeutic targets in diabetic foot ulceration
Source: Diabetol Metab Syndr. 2025 Aug 1;17:306. doi: 10.1186/s13098-025-01880-9 (PMC12315281; doi:10.1186/s13098-025-01880-9)
Supplement: Supplementary file 11 — Supplementary Material 11 [file 13098_2025_1880_MOESM11_ESM.docx]

### Supplementary Table S11 mRNA-TF Interaction Network Nodes

| mRNA | TF |
| --- | --- |
| FSTL1 | ELF1 |
| FSTL1 | ERG |
| FSTL1 | ESRRA |
| FSTL1 | GABPA |
| FSTL1 | NFYB |
| FSTL1 | NRF1 |
| CPTP | ELF1 |
| CPTP | ERG |
| CPTP | ESRRA |
| CPTP | GABPA |
| CPTP | NFYB |
| CPTP | NRF1 |
| FSTL1 | SPI1 |
| FSTL1 | TEAD4 |
| FSTL1 | CEBPA |
| FSTL1 | CEBPB |
| HDAC3 | EGR1 |
| HDAC3 | ELF1 |
| HDAC3 | EP300 |
| HDAC3 | ERG |
| HDAC3 | FOS |
| HDAC3 | FOSL1 |
| HDAC3 | FOSL2 |
| HDAC3 | JUN |
| HDAC3 | JUNB |
| HDAC3 | JUND |
| HDAC3 | MAX |
| HDAC3 | MYC |
| HDAC3 | NRF1 |
| HDAC3 | POLR2A |
| HDAC3 | RAD21 |
| HDAC3 | REST |
| HDAC3 | RUNX1 |
| HDAC3 | SMARCA4 |
| HDAC3 | SMC3 |
| HDAC3 | SPI1 |
| HDAC3 | STAG1 |
| HDAC3 | STAT3 |
| HDAC3 | CTCF |
| PINK1 | E2F6 |
| PINK1 | EGR1 |
| PINK1 | HNF4A |
| PINK1 | MAX |
| PINK1 | MYC |
| PINK1 | NRF1 |
| PINK1 | SP1 |
| ULK1 | EGR1 |
| ULK1 | NRF1 |

TF：Transcription Factors
